# Joint Cognitive Models Reveal Sources of Robust Individual Differences in Conflict Processing

**DOI:** 10.1007/s42113-026-00263-1

**Published:** 2026-03-19

**Authors:** M. Fiona Molloy, Taraz G. Lee, John Jonides, Han Zhang, Jacob Sellers, Andrew Heathcote, Chandra Sripada, Alexander S. Weigard

**Affiliations:** 1 Department of Psychiatry, University of Michigan, 4250 Plymouth Road, Ann Arbor, MI 48109 USA; 2 Department of Psychology, University of Michigan, Ann Arbor, MI USA; 3 Department of Psychological Methods, University of Amsterdam, Amsterdam, The Netherlands; 4 School of Psychology, University of Newcastle, Newcastle, Australia

**Keywords:** Conflict task, Joint modeling, Hierarchical Bayesian, Linear ballistic accumulator model, Individual differences

## Abstract

Experimental manipulations in conflict tasks, e.g., the Stroop, Flanker, and Simon tasks, lead to systematically poorer performance in “incongruent” conditions that feature stimuli that contradict task goals. However, substantial recent debate surrounds whether individual differences in conflict task behavior reflect reliable, trait-like mechanistic processes. Much prior work uses difference scores, contrasting performance between incongruent and congruent trials to index conflict suppression ability, but recent work demonstrates these scores exhibit poor psychometric properties. Formal cognitive process models suggest that individual differences in conflict suppression are driven by task-general processes, as opposed to processes specialized for conflict. However, this prior work separately models cognitive process parameters and their covariation, which fails to adequately account for measurement error. Here, we model distinct mechanisms of conflict task performance and their covariance simultaneously using hierarchical Bayesian joint modeling methods for the first time which improves individual estimation and accounts for error. We fit the conflict linear ballistic accumulator model (LBA) to two large datasets containing multiple conflict tasks and test-retest sessions, and an additional large dataset containing a conflict task and simple perceptual decision-making task. First, within conflict tasks, we found moderate test-retest reliability for both conflict-specific processing mechanisms, and, to a larger degree, task-general mechanisms. Second, task-general, but not conflict-specific, mechanisms were correlated across different conflict tasks. Third, these task-general mechanisms were correlated between conflict tasks and a simple decision-making task without conflict suppression demands. Overall, we found robust individual differences in computational mechanisms underlying general decision-making, but not mechanisms specific to conflict processing.

**Supplementary Information:**

The online version contains supplementary material available at 10.1007/s42113-026-00263-1.

## Introduction

To efficiently and accurately execute complex goal-directed responses, an agent must selectively identify goal-relevant evidence in the environment. Sometimes, the environment additionally contains irrelevant information that promotes incorrect responses, making accurate and efficient task performance more difficult. Decision-making with this type of conflicting evidence (i.e., goal-relevant versus goal-irrelevant) is often studied using conflict tasks such as the well-known Flanker (B. A. Eriksen & Eriksen, 1974), Simon (Simon & Rudell, 1967), and Stroop tasks (Stroop, 1935). These tasks have been widely used in experimental psychology and have greatly enhanced our understanding of cognitive control processes (Eriksen, 1995; Hommel, 2011; MacLeod, 1991).

However, insights from this experimental literature have been difficult to translate to the study of individual differences. Individual differences in conflict resolution ability have long been hypothesized to reflect a construct termed “attentional control” (Burgoyne et al., 2023; Mashburn et al., 2024; Oberauer, 2024; von Bastian et al., 2020), defined as an individual’s ability to control their attention in the service of goal-directed behavior. This ability is thought to be selectively or preferentially engaged in the incongruent condition (in which goal irrelevant evidence conflicts with the goal relevant response) compared to in the congruent condition (in which goal irrelevant evidence facilitates the goal relevant response). Thus, individual differences in attentional control are typically quantified using difference (or subtraction) scores in which the mean response time (RT) for congruent trials is subtracted from the mean response time for incongruent trials.

Although this index is simple to compute and reflects conflict effects that have been widely replicated across studies on a group-level, its use in studying individual differences has proved challenging (Draheim et al., 2019) due to its poor psychometric properties. In particular, multiple large studies employing diverse batteries of conflict tasks consistently find that difference scores exhibit poor test-retest reliability (Enkavi et al., 2019; Hedge et al., 2018). Additionally, difference score measures fail to correlate across conflict tasks, including those that should be theoretically related to each other (Frischkorn et al., 2019; Karr et al., 2018; Rey-Mermet et al., 2019), even after accounting for measurement error or suboptimal conditions and modeling trial-to-trial variability, instead of aggregating across trials (Rouder & Haaf, 2019).

Formal cognitive models provide a promising approach to measuring conflict resolution mechanisms that may also address some of the preceding problems. Unlike difference scores in behavioral summary statistics (RT, accuracy), formal cognitive models provide a rich and detailed description of cognitive operations during a task, explaining within-trial dynamics of decision-making using precise and interpretable mechanisms. Formal cognitive models have been informative across many domains of psychology and neuroscience, including applications to language, memory, and attention (Mulder et al., 2014; Ratcliff et al., 2016). In addition to the benefit of dissociating mechanistic contributors to behavior, cognitive models may also improve measurement reliability by integrating a comprehensive account of behavior, including the RT distributions (Haines et al., 2025) and accuracy in a theoretically guided way (Donkin & Brown, 2018).

Evidence accumulation models, which assume that individuals select responses by gradually accumulating noisy evidence from stimuli, are a well-validated class of cognitive models that have been widely applied to tasks that index a variety of complex behaviors (Heathcote & Matzke, 2022). Key decision-making mechanisms can be incorporated into evidence accumulation models to account for the impact of irrelevant information on conflict tasks (Hübner et al., 2010; Ulrich et al., 2015; White et al., 2011). Several such frameworks are based on the diffusion decision model (DDM), which posits that evidence gathered from a stimulus informs a single “total evidence” variable that drifts between two boundaries representing each possible response, and that responses are selected when the evidence process crosses one of the boundaries (Ratcliff, 1978; Ratcliff et al., 2016).

Although the various “conflict DDM” frameworks have slightly different architectures, they all share the assumption that conflicting information impacts the “drift rate” at which the evidence accumulation process drifts toward the correct response boundary. Conflict is posited to cause an initial deflection of the evidence accumulation process toward the boundary favored by the task-irrelevant information early in the evidence accumulation process. The influence of this conflicting evidence wanes as accumulation continues and becomes replaced by the influence of task-relevant evidence that is operationalized as a standard DDM parameter for drift toward the correct response boundary. This temporal dynamic can explain findings of fast error RTs in some conflict paradigms. Although these conflict models provide compelling accounts of the transient influence of conflicting information on processing, their application to empirical data is complex due to the lack of a closed form likelihood function and problematic parameter recovery properties (White et al., 2018).

Nonetheless, there is evidence that parameters from evidence accumulation models could improve test-retest reliability relative to standard difference scores in conflict tasks. Hedge et al. (2022), who used the diffusion model for conflict tasks (DMC; Ulrich et al., 2015), found that parameters that index the early influence of conflicting information on processing displayed reasonable test-retest reliability, in addition to the reasonable test-retest reliability found for drift rate and boundary separation. However, they found no correlation in conflict parameters across different conflict tasks in multiple datasets, suggesting the absence of a general ability for the suppression of conflicting information. Instead, they found that more task-general processes indexed by evidence accumulation models, including individuals’ general rate of goal-relevant evidence accumulation (drift rate parameter) and response caution (boundary separation) did show strong concordance across the tasks.

These findings are consistent with an emerging cognitive modeling literature finding that individual differences in performance on executive or cognitive control tasks are not attributable to specialized processes that are only active on these tasks and are instead largely driven by task-general processes. For example, Weigard et al. (2021) combined DDM parameter estimates with a structural equation modeling approach across seven difference cognitive control tasks to demonstrate that, although difference scores did not cohere into latent factors, performance across conditions thought to require cognitive control (e.g., incongruent trials in conflict tasks) and those thought not to (e.g., congruent trials) were both largely driven by the same factor for individuals’ general efficiency of evidence accumulation (EEA). Such a general factor has been widely replicated across diverse samples (Lerche et al., 2020; Schmiedek et al., 2007; Schubert & Frischkorn, 2020; Vermeent et al., 2024; Weigard & Sripada, 2021; Hedge et al., 2022) and appears to largely explain performance in both relatively simple tasks as well as tasks previously thought to measure specialized executive functions (Löffler et al., 2024).

This growing line of work suggests that individual differences in conflict task performance may be largely driven by task-general processes that are active across both congruent and incongruent conditions. In turn, such work suggests that suppression of irrelevant information in the incongruent trials of specific conflict tasks may instead reflect an array of idiosyncratic processes that are engaged in these specific conditions, rather than reflecting a trait-like conflict-resolution construct. Thus, this work challenges a foundational “conflict-specific” assumption of most extant work on attentional control, which says that trait-like cognitive mechanisms for selecting task-relevant information are selectively or preferentially engaged in task conditions that include conflicting task-irrelevant information (i.e., the incongruent condition of conflict tasks).

Indeed, we posit that this “conflict-specific” assumption is incorrect. Instead, we propose an alternative “task-general” view that cognitive mechanisms for selecting task-relevant information are operative *to an equal degree* across conditions that include conflicting task-irrelevant information (i.e., incongruent conditions of conflict tasks) and those that do not (i.e., congruent conditions of conflict tasks, as well as simple tasks without any manipulation of conflict). We propose that the non-selective nature of these mechanisms helps to explain consistent findings of a task-general EEA factor in prior studies of conflict tasks.

However, our rejection of the conflict-specific assumption, which says that conflict task manipulations selectively or preferentially engage attentional control mechanisms, may be premature if model parameters that index these putatively selective mechanisms are not being estimated under optimal conditions (such as trial-to-trial variability and measurement error). Hierarchical Bayesian methods offer several key estimation advantages over the approaches used in prior applications of conflict DDMs, including Hedge et al. (2022), which relied on point estimates of conflict model parameters to uncover cross-task correlations in conflict processes. Rouder and Haaf (2019) demonstrated that hierarchical Bayesian models yield improved within-task test-retest reliability of summary statistic (e.g., RT) difference scores from conflict tasks. This mathematical framework also more accurately estimates parameters for individuals (James & Stein, 1961), and its key property, shrinkage, can allow for more accurate estimates overall compared to point estimates (Efron & Morris, 1977; Lehmann & Casella, 1998). Additionally, using point estimates can be problematic when doing post-hoc correlations, such as correlating model parameters across tasks after separately fitting the models, due to attenuation (Matzke et al., 2017; Rouder & Haaf, 2019). Attenuation results in the correlation between two measures being much lower than the true value due to uncertainty and/or measurement error. Hence, the reported low correlations of measures of conflict suppression ability across different conflict tasks could be underestimated due to attenuation.

One approach for addressing the problems raised by attenuation is to directly model the measurement uncertainty within hierarchical Bayesian models (Matzke et al., 2017). Instead of relying on point estimates of the correlation (which will be downwardly biased due to attenuation), hierarchical Bayesian models more appropriately represent measurement uncertainty in the spread of the posterior, e.g., affecting only the credible interval, not the central tendency. However, even in a Bayesian framework, when using RT difference scores, Rouder and Haaf (2019) still found no evidence of a common conflict effect across conflict tasks (specifically Flanker and Stroop tasks). Importantly, though, Rouder and Haaf did not use cognitive models that allow measurement of specific mechanisms that underlie behavior on conflict tasks, which, as discussed above, have been shown in non-Bayesian frameworks to increase reliability and interpretability of conflict task measures (Eisenberg et al., 2019; Weigard et al., 2021).

In the present study, we propose to integrate the strengths of Bayesian methods and cognitive modeling within a single joint modeling framework. Joint modeling simultaneously estimates parameters from different datasets, tasks, and/or modalities. Similar to Bayesian methods and cognitive modeling in general, joint modeling frameworks have been found to lead to more precise and reliable individual estimates (Forstmann et al., 2008; Kvam et al., 2021; Wall et al., 2021). Although there are different types of joint models (Palestro et al., 2018), here we utilize a covariance joint model (Turner et al., 2013). In this approach, separate models are connected via a linking function, often a multivariate normal distribution. Then, each model’s parameters and the covariance between the different model’s parameters are simultaneously estimated. Covariance joint models have been utilized to investigate a wide variety of behavioral tasks to explore common latent processes across different domains, including visual search, stop-signal, and match to memory tasks (Wall et al., 2021) and delay discounting and Cambridge gambling tasks (Kvam et al., 2021).

We selected the conflict linear ballistic accumulator model (LBA) to model conflict in our analyses due to evidence of problematic parameter recovery of conflict DDMs (White et al., 2018). The conflict LBA extends the standard LBA model (Brown & Heathcote, 2008) to include key mechanisms to account for conflict effects. The standard LBA is specified as a race between an accumulator for each choice, where a decision is made once the accumulator crosses a threshold, *b*. Evidence is accumulated at a constant rate, where the rate in a given trial is sampled from a normal distribution with mean *v*, analogous to a drift rate. EEA is measured in this model as the difference between the drift rate for the accumulator matching the correct response on a given trial and the drift rate for accumulators mismatching the correct response. Other parameters include *A*, starting point variability, and *t*_*0*_, non-decision time.

**Fig. 1:**
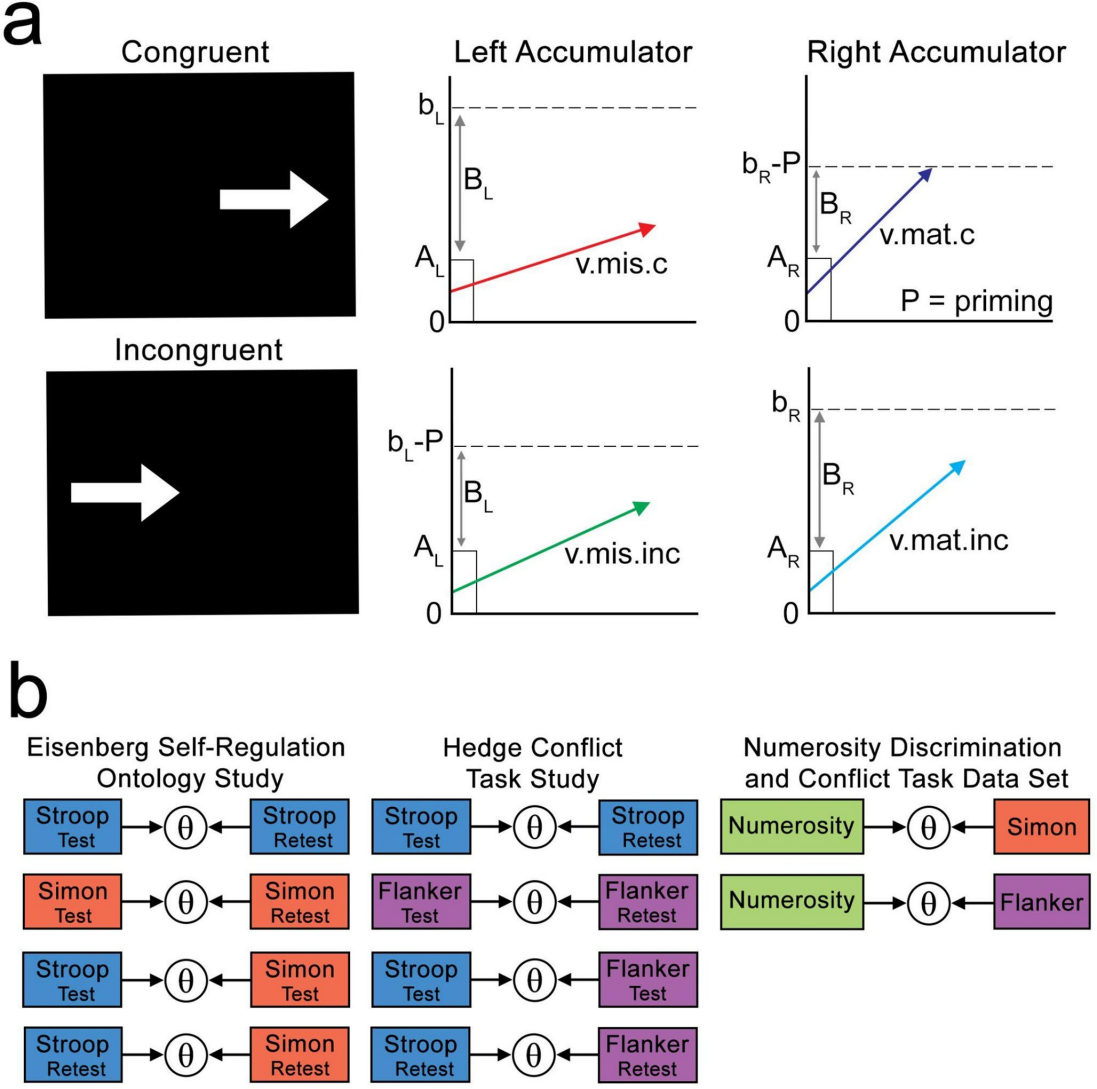
Schematic of conflict LBA and joint models. **a**. Conflict LBA model of a standard Simon task,where participants must press the left or right arrow key to report which side of the screen thearrow is shown. The top row shows an example congruent (con) trial and the bottom row showsan example incongruent (inc) trial. A, starting point variability; b, threshold; v, mean drift rate;mat, accumulator matching the stimulus; mis, accumulator mismatching the stimulus; B=b-P, threshold for the primed accumulator; L, left accumulator; R, right accumulator. Note that threeparameters are estimated to describe drift rate: 1) overall evidence quality (main effect of latentmatch or v.mat – v.mis), 2) Δ evidence quantity (main effect of congruency or v.inc – v.con), and3) Δ evidence quality (interaction effect of latent match and congruency). **b**. Summary of jointmodels fit showing each pair of task/sessions in each model

Conflict is incorporated through two effects on key parameters (Fig. 1a). First, the early and transient effect of irrelevant information is assumed to shift the start point of the associated accumulator higher (i.e., as an early priming effect). This is analogous to parameters from conflict DDMs that assume an early deflection of the decision variable toward the boundary favored by irrelevant information. As start point increases in accumulator models are mathematically equivalent to threshold decreases, this effect is implemented through the threshold parameter in the conflict LBA, usually resulting in a lower threshold for accumulators favored by task-relevant information (i.e., the correct response accumulator on congruent trials and incorrect response accumulator on incongruent trials). Second, ongoing effects of task-irrelevant information on the evidence accumulation process are modeled by allowing the drift rates of the correct and incorrect response accumulators to differ by congruency condition. Past work (Weigard et al., 2019; Weigard, Huang-Pollock, Brown et al., 2018a; Weigard, Huang-Pollock, Heathcote et al., 2018b) has shown that the incongruent condition is characterized by both poorer *quantity* of evidence (slower drift rates for the accumulators that both match and mismatch the correct response) and poorer *quality* of evidence (slower drift rate for the accumulator matching the correct response verses the mismatching accumulator). Note that the incongruent condition is therefore characterized by higher thresholds and slower drift rates, which can both yield longer RTs characteristic of conflict effects. In this way, the conflict LBA provides a principled dissociation of distinct aspects of conflict processing while maintaining key advantages of the LBA model relative to conflict DDMs, including a closed form likelihood function and good parameter recovery.

Here, we use the conflict LBA model within a hierarchical Bayesian joint modeling framework to gauge the reliability and cross-task relevance of individual differences in cognitive mechanisms underlying performance on conflict tasks. First, within two large datasets from Hedge et al. (2018) and Eisenberg et al. (2019) containing test and retest sessions for the Simon, Stroop, and Flanker tasks, we determine which conflict LBA model mechanisms are necessary to explain choice and response time in each task and sample. Second, using within-task joint modeling, we report the test-retest reliability of conflict LBA model parameters in these tasks. Third, using cross-task joint modeling, we determine which mechanisms, if any, are correlated across different conflict tasks.

Finally, given prior findings that the only parameters that show correlations across tasks represented general mechanisms that are likely involved in performance across many tasks beyond conflict paradigms (e.g., Weigard, 2021), we extended our study to simple decision-making tasks that are traditionally assumed to not involve conflict suppression. To do so, we again use cross-task joint modeling to test whether common decision-making mechanisms in conflict and simple perceptual decision-making tasks are correlated using an additional dataset of individuals who completed a conflict task and a numerosity discrimination task.

Overall, we leverage a joint modeling framework that directly accounts for measurement error to assess the trait-like properties of two categories of mechanisms that potentially drive behavior on conflict tasks: (1) task-general mechanisms that drive performance equally across the congruent and incongruent conditions of conflict tasks (e.g., EEA, response threshold), and (2) conflict-specific mechanisms selectively engaged in the incongruent condition (e.g., early priming effects on evidence accumulation and drift rate differences between conditions). Findings that the latter (conflict-specific) mechanisms display both reliability and high cross-task correlations would support the traditional view that an attentional control mechanism is selectively or preferentially engaged in the presence of conflict. In contrast, findings that only the former (task-general) mechanisms display both reliability and high cross-task correlations would support our alternative hypothesis that mechanisms for selectively gathering task-relevant information are operative across diverse task conditions including those that do and do not involve conflict.

## Methods

A schematic of the conflict LBA model and summary of the joint models fit here are shown in Fig. 1.

### Datasets

We fit the conflict LBA model to three samples of individuals who completed conflict and related behavioral tasks as part of larger studies from Eisenberg et al. (2019), Hedge et al. (2018), and a study of individuals who completed forced-response and free-response versions of conflict tasks along with a basic perceptual decision task (numerosity discrimination). The samples and conflict tasks will be described briefly below.

#### Eisenberg Self-Regulation Ontology Study

Beginning with the data from Eisenberg et al. (2019), we modeled two conflict tasks that were part of a larger battery of tasks thought to be relevant to self-regulation: Stroop and Simon. In the Simon task, participants were asked to press a button (left or right arrow) depending on the color of a box (red or blue) presented on either the left or right side of the screen. There were 2 conditions: congruent, in which the side of the screen matched the direction of the response key; and incongruent, in which the stimuli were presented on the opposite side of the direction of the response key. In the Simon task, location strongly primes the associated response. Participants completed 120 trials (60 per condition), including 20 practice trials that we included in modeling to maximize the number of trials available. In the Stroop task, participants were asked to report the font color (red, green, or blue) of the presented word (3 total responses). In the congruent condition, the font color matched the written word; in the incongruent condition, the font color did not match the written words. In the Stroop task, the word reading is assumed to be automatic and primes the associated response. In each session, participants completed 120 trials (60 per condition), including 24 practice trials.

A total of 522 individuals completed the task battery (test sample size pre-exclusion = 522), and 150 of those individuals completed a second session (retest sample size pre-exclusion = 150). The time in between sessions was between 60 and 228 days. For preprocessing in both tasks, omissions and response times shorter than 300 ms were removed, following previous work using the conflict LBA (Weigard et al., 2019). Individuals with less than chance accuracy were removed (removing one individual from the Simon test session with an accuracy less than 50%). All mean accuracies were above 95% (mean accuracy for Simon test: 95.5%; Simon retest: 95.9%; Stroop test: 95.9%; Stroop retest: 95.6%). The mean number of included trials per subject was approximately 118 (mean total trials for Simon test: 118.6 [range: 49–120]; Simon retest: 118.8 [98–120]; Stroop test: 117.1 [33–120]; Stroop retest: 117.9 [103–120]). The final sample included 521 individuals for the Simon test session, 522 individuals for the Stroop test session, and 151 individuals for both the Simon and Stroop retest sessions.

#### Hedge Conflict Task Study

In the data from Hedge et al. (2018), we focused on two conflict tasks: Stroop and Flanker. Participants completed these tasks as part of a larger battery across two sessions (test and retest) 3 weeks apart. In the Flanker task, participants were asked to report the direction (left or right) of a central arrow. There were a total of 3 conditions: congruent, in which the central arrow was surrounded by arrows pointing in the same direction; incongruent, in which the central arrow was surrounded by arrows pointing in the opposite direction), and neutral (in which the arrow was surrounded by straight lines). Each participant completed 720 trials total (240 per condition) per session. In the Stroop task, participants were asked to report the font color of a word presented. There were 4 response options: red, blue, green, and yellow. Similarly to the Flanker task, there were 3 conditions, congruent (written word was the font color), incongruent (written word had a different font color), and neutral (the written word was not a color, but was instead “lot”, “ship”, “cross”, or “advice”). Additionally, each participant completed 720 total trials (240 per condition).

For both the Flanker and the Stroop, only trials with a RT greater than 300 ms and less than 3000 ms were included. Neutral trials were removed to allow for direct comparison with the other datasets and to reduce the total number of parameters in the conflict LBA by removing the third condition. For the Flanker task, the mean number of trials after preprocessing was 458.7 trials in the test session (range: 148–480) and 433.1 in the retest session (range: 142–480). All individuals in the Flanker test and retest sessions had an accuracy of greater than 50%, with a mean accuracy of 91.5% and 90.1% in the test and retest sessions, respectively. For the Stroop task, the mean number of trials was 479 in the test session (range: 456–480) and 476.3 in the retest session (range: 388–480). All individuals in the Stroop test and retest sessions had an accuracy > 50%, with a mean accuracy of 92.7% in the test session and 91.5% in the retest session. The final sample sizes per task and session were *N* = 106 for Flanker test, *N* = 107 for Flanker retest, and *N* = 103 for Stroop test and retest.

#### Numerosity Discrimination and Conflict Task Data Set

The final dataset was drawn from a study focused on comparing conflict measures from forced-choice (Lee et al., 2024) and free-response (e.g., the standard version) versions of the Simon and Flanker tasks that also included a basic perceptual decision task (numerosity discrimination). We modeled data from the free-response conflict and numerosity paradigms and note that comparisons with forced-response measures will be reported elsewhere. Participants (*n* = 234; mean age = 27.6, age range = 18–35, 112 females) were recruited using the Prolific online research platform and were asked to complete three sessions of data collection over a web browser: (1) a forced-response conflict task, (2) a free-response conflict task, and (3) the numerosity discrimination task. Every participant in the study completed the numerosity task as well as one of each of the conflict tasks (Simon *N* = 116, Flanker *N* = 119).

The Flanker task and Simon tasks both consisted of 420 total trials (50% congruent, 50% incongruent condition). Possible responses were “w” or “p” indicating a “left” or “right” response respectively. Trials were removed if the RT was missing or shorter than 300 ms. All accuracies in both tasks were above 50%, and the mean accuracy was 96.7% for the Flanker task and 95.1% for the Simon task. Following data cleaning, no individuals were removed, and the average total trials was 404.9 for the Flanker task (range: 315–417) and 400 (range: 223–415) for the Simon task.

The numerosity task contained a total of 200 trials in which participants were presented with arrays of asterisks on a 10 × 10 grid and asked to report if there were “few” (< 50) or “many” (> 50) stimuli. The task contained both hard (41–45 or 55–59 asterisks) and easy (31–35 or 65–69 asterisks) condition trials that were randomly interspersed. The preprocessing steps were similar to the above tasks. Specifically, any missing response times were excluded, as were RTs shorter than 300 ms. Seven individuals had an accuracy of lower than 50% and were removed. Following these exclusion criteria, we additionally removed 12 individuals (note there is overlap with the individuals with low accuracy) who lost more than 50% of the trials, i.e. only keeping individuals with 100 trials or greater. The final numerosity dataset included *N* = 222 individuals, with a mean accuracy of 90.18%.

### Model Specification

As described in the introduction, the conflict LBA is an extension of the linear ballistic accumulator model (LBA) developed for conflict tasks. The framework contains the main components present in evidence accumulation models, including response threshold (B), drift rate (v), starting point variability (A), and non-decision time (t0). Two key mechanisms are added to describe behavior in conflict tasks: (1) a priming mechanism that accounts for the early transient influence of task-irrelevant information on evidence accumulation, implemented by the threshold parameter, and (2) the persistent influence of task-irrelevant information on processing throughout the trial implemented by drift rate parameters.

Rate variability (sv) and rate intercept (v, using EMC2, the model fitting package, nomenclature) parameters were fixed in all models (sv = 1 and v = 1) to address issues in identifiability, particularly related to the low error rates in the data (Donkin et al., 2009; Evans, 2020; Lüken et al., 2025). Note that parameterizations are specified in a linear modeling framework, where v is an intercept parameter for drift (overall rate “quantity”, or the “urgency” of responding regardless of stimuli, Strickland et al., 2018, Miletić et al., 2021). All models have a “general drift” or evidence quality parameter, which is the effect of “latent match” or the difference between the rate of the accumulator matching the correct response and the rate of mismatching accumulators (which were assumed to have the same rate when there was more than one). This parameter is analogous to the drift rate in the DDM, varies across conditions, and quantifies the ability to identify the correct response to the stimuli (Strickland et al., 2018). To determine which conflict LBA model mechanisms are necessary to explain choice and RT in each sample, four models were fit for each dataset and task. The null model is a standard LBA and does not contain any conflict-specific mechanisms (4 parameters: overall evidence quality, threshold, starting point, non-decision time). Model E has two additional parameters to specify an effect of conflict on evidence accumulation. We refer to this model as E, as the parameters correspond to the difference (Δ) in evidence quantity (the main effect of congruency on drift rates in the linear model) and evidence quality (an interaction effect of drift match and congruency). Model P has an additional parameter to specify an effect of priming on threshold. Finally, the full model (pictured in Fig. 1a) contains both conflict mechanisms implemented in Model E and Model P. The full model has a total of 7 parameters. Four are general decision-making parameters (overall evidence quality, threshold, starting point, and non-decision time) and three are conflict-specific parameters (Δ evidence quantity, Δ evidence quality, and threshold priming).

Model comparison was also conducted for the numerosity task to select an appropriate specification of the LBA model for the final analyses comparing numerosity discrimination and conflict model parameters. Four models were again fit, with similar specifications to the conflict model, but reflecting features unique to the numerosity task. The models include a null model (standard LBA), an evidence accumulation effect model with two additional parameters for the effect of trial difficulty (hard and easy) on evidence accumulation, a threshold effect model with an additional parameter to the null model for the effect of few vs. many asterisk stimuli on adjusting thresholds to address possible response biases (Weigard, Huang-Pollock, Brown, et al., 2018a), and a full model containing both the evidence accumulation and threshold effect model mechanisms. The full model contains 7 parameters. Four are general decision-making parameters (overall evidence quality, threshold, starting point, and non-decision time) and 3 are parameters specific to the numerosity task (Δ evidence quantity or the main effect of difficulty, Δ evidence quality or the interaction effect of latent match and difficulty, and threshold adjustment for response biases). The full model was selected as the winning model as it had the minimum deviance information criterion, DIC (DIC difference from the best model: null: 6,808, evidence accumulation effect model: 1,998, threshold effect model: 4,922, full model: 0).

### Model Fitting and Selection

We fit all models using the “Extended Models of Choice” (EMC2) R package, version 0.0.0.9000 (Stevenson et al., 2024). Models were fit in a hierarchical Bayesian framework, constructing a hierarchy across participants to simultaneously estimate individual and group-level parameters. Unless specified otherwise, EMC2’s default sampler settings and priors were used.

A model was considered converged when the Gelman-Rubin statistic, or potential scale reduction factor, of all parameters (including the individual-level, group-level, and correlations between parameters) were less than 1.1, indicating sufficient mixing of all (3) chains (Gelman & Rubin, 1992). If a model did not converge with 40 sampling steps (4,000 iterations), the next stage of sampling increased the number of particles of the sampler (or particle factor) to 100 and decreased the target acceptance probability to 0.6, both of which allow for a wider search of the likelihood space, and was run until the model converged.

Model fit, penalizing for complexity, was assessed using the deviance information criterion, or DIC (Spiegelhalter et al., 2002), where the preferred model for a given task and dataset has the lowest value.

### Joint Model Specification and Analysis

Once the preferred models were identified separately for each task, we built a series of joint models to answer three main questions about the stability and generalizability of conflict task mechanisms. The tasks and dataset in each joint model are summarized in Fig. 1b. First, we examined the test-retest reliability of model parameters within a given task. Four models were fit, and the total number of individuals within each joint model (e.g., completed both the test and retest session) is reported. For the Eisenberg et al. sample, we fit within-task joint models for the Stroop (*N* = 150 individuals) and Simon (*N* = 150) tasks. For the Hedge et al. sample, within-task joint models were fit for the Stroop (*N* = 103) and the Flanker (*N* = 106) tasks.

Second, we examined whether these model parameters correspond to each other across different conflict tasks. In other words, within the same session, how linearly related are people’s decision-making dynamics across these different tasks that are thought to measure the same construct? For the Eisenberg et al. (2019) dataset, joint models looking at the covariance structure between the Stroop and Simon task were fit separately for the test (*N* = 521) and retest (*N* = 151) sessions. Similarly, for the Hedge et al. (2018) sample joint models were fit to the Flanker and Stroop data for the test (*N* = 103) and retest (*N* = 103) sessions.

Lastly, we quantified the covariance between decision-making mechanisms of conflict tasks and those in a numerosity discrimination task, a basic perceptual decision task thought to have no explicit demands on cognitive control. Using the numerosity dataset, we fit a total of two joint models, one for the individuals who completed the Flanker and numerosity tasks (*N* = 101), and one for the individuals who completed the Simon and numerosity tasks (*N* = 105).

## Results

### Identifying Important Conflict Mechanisms Using Model Fit

**Fig. 2:**
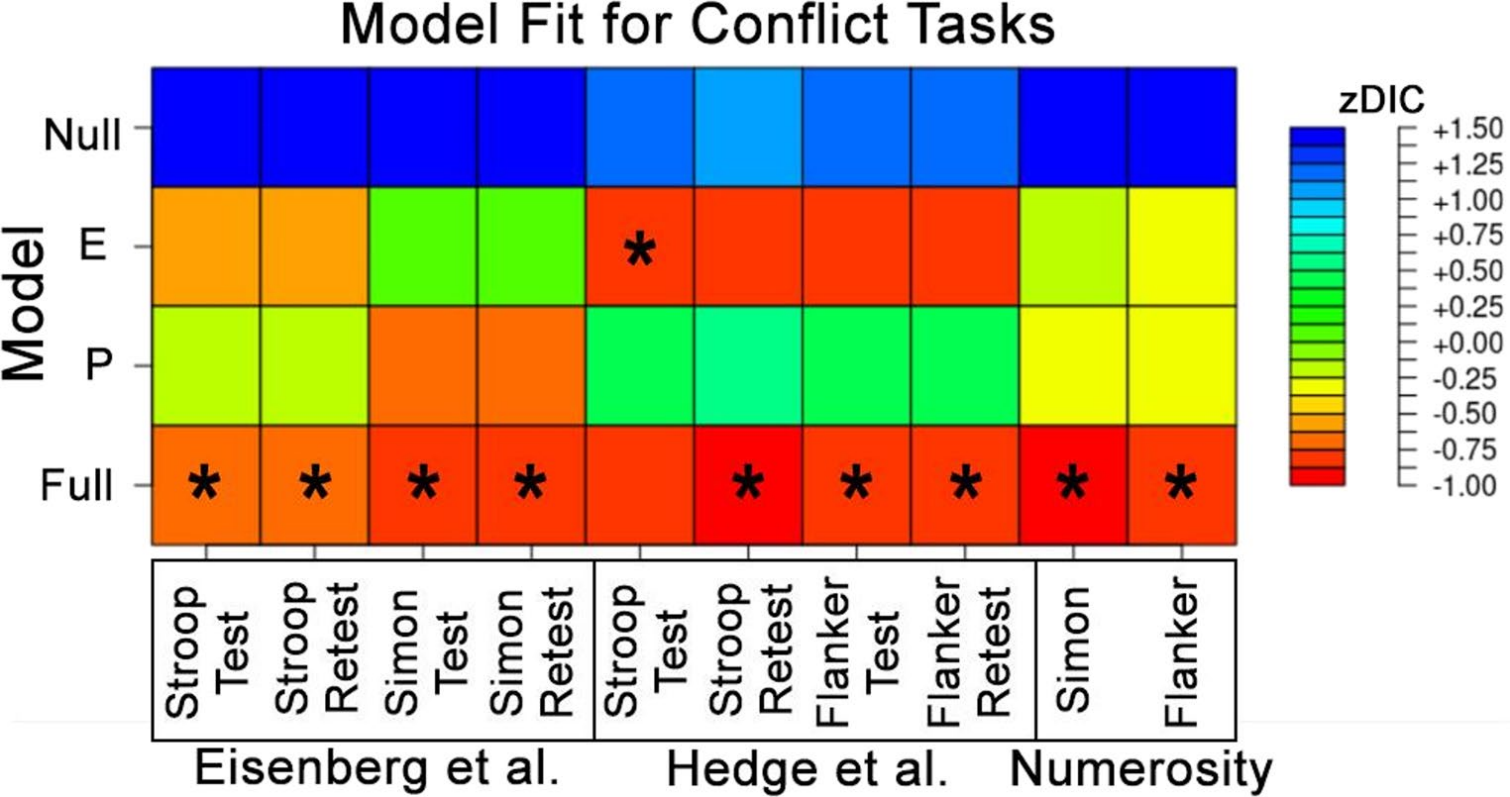
Model comparison results. Asterisks indicate the winning model for each dataset/task. Model fit penalized by complexity is quantified here using the deviance information criterion, or DIC (Spiegelhalter et al., 2002). The model with the lowest DIC for each task (columns) is considered the winning model (indicated by asterisks). For plotting purposes, DIC is z-scored within each column

To begin, we investigated which conflict LBA model mechanisms are important for explaining choice and response time behavior within each task (Flanker, Stroop, and Simon) and dataset. We evaluated four candidate models with potential conflict effect explanations including parameters for congruency’s transient impact on early evidence accumulation and persistent impact on evidence accumulation throughout the trial. The model fit metrics (DIC), the degree of fit penalized by complexity, are plotted in Fig. 2, for each model (row) and task within each sample (columns). To allow for comparison across tasks and datasets, the DIC values in each column were z-scored. The winning model (lowest DIC) is denoted by an asterisk.

Overall, conflict task data were best explained by the full model variant with mechanisms both allowing the condition of the trials (congruent or incongruent) to affect drift rate and allowing priming to affect the threshold. In the Simon and Flanker tasks for all datasets, the full model was preferred. For the Stroop task, the full model was preferred for the test and retest sessions from Eisenberg et al., and for the retest session from Hedge et al. However, model E was preferred for the test session from Hedge et al., though the relative DICs for model E and the full model were highly similar (zDIC for model E: −0.824; zDIC for full model: −0.821). In other cases as well, such as the Flanker task from Hedge et al., model E and the full model had similar performance. Likely, both models fit similarly well, and the winning model depends on the penalization for complexity (see Supplementary Figure S1 for a corresponding plot of the zBPIC scores, which has a larger penalty for complexity). For the purposes of the joint modeling, we chose to follow the conclusions of the DIC and select the full model for each task. This allows us to investigate the reliability and task-generality of the priming process in all datasets. Model fit plots, including fit to the delta functions, for the full models are shown in Supplementary Figures S2-S9.

### Conflict Task Reliability

**Fig. 3:**
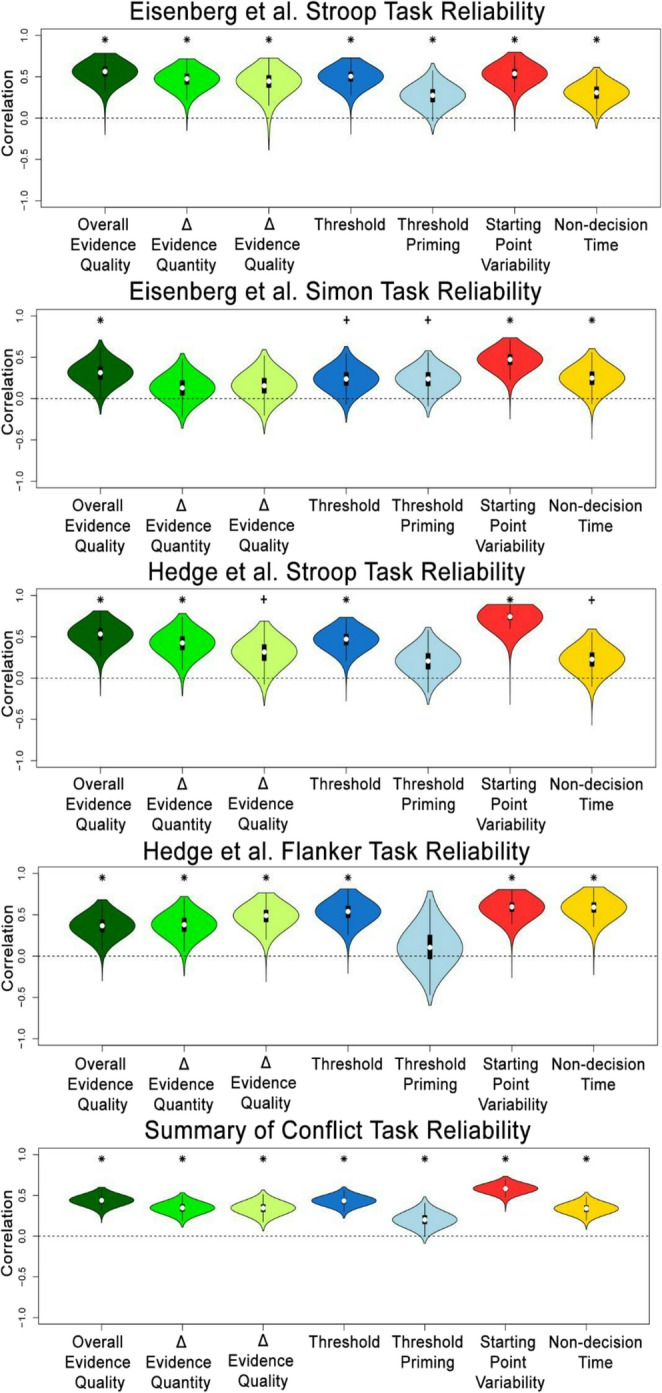
Posterior distributions of test-retest correlations within conflict tasks. * indicates the 95% credible interval does not contain 0, and + indicates the 89% credible interval does not contain 0

Before looking at mechanisms common across different conflict tasks, we sought to confirm that model parameters are stable within the same task across sessions. Through joint modeling, we can estimate the covariance structure of the parameters of the conflict LBA between test and retest sessions of a given conflict task. Figure 3 displays violin plots of the posterior distributions of the test-retest correlations for each parameter and task within the Eisenberg and Hedge datasets. In all cases, the means of the test-retest correlation posteriors were positive (see Supplementary Table S1 and Table S2 for mean, 95% credible interval [C.I.] limits, and standard deviations for all posteriors presented in Fig. 3).

General evidence quality displayed posterior median test-retest correlations between 0.31 and 0.55 and the 95% CIs were consistently greater than 0. Conflict effects on evidence quality (median range: 0.15–0.48) and evidence quantity (0.13–0.47) showed generally lower reliability and CIs included 0 for the Eisenberg Simon task for both conflict effects on evidence. A similar pattern was observed in the threshold parameters. Threshold test-retest correlations were consistently greater than 0 with comparable reliability (0.23–0.53) to overall evidence quality. Again, the conflict-task specific mechanism, threshold priming, had lower overall reliability (0.11–0.27), particularly for the Hedge Stroop and Flanker tasks, where both CIs contained 0. The reliability was noticeably lower in threshold priming in the Flanker task, where the posterior was wide and centered very close to 0 (median: 0.11, standard deviation: 0.22). Finally, in all tasks, the starting point variability reliability was high (0.47–0.74), and while the non-decision time test-retest correlations were lower (0.22–0.58), all CIs were consistently above 0.

In sum, most parameters displayed some evidence of reliability across timepoints, including parameters for conflict-related processing differences, with the test-retest reliability of parameters involved in general performance across conditions (overall evidence quality, threshold) being only slightly greater than that of conflict parameters, as shown by the meta-analytic posteriors in the last row of Fig. 3. Although reliability estimates from these single task parameters are not optimal for applications to individual differences studies, they nonetheless suggest some level of stable trait-like variance for each of the parameters.

### Cross-task Correlations in Conflict Mechanisms

**Fig. 4:**
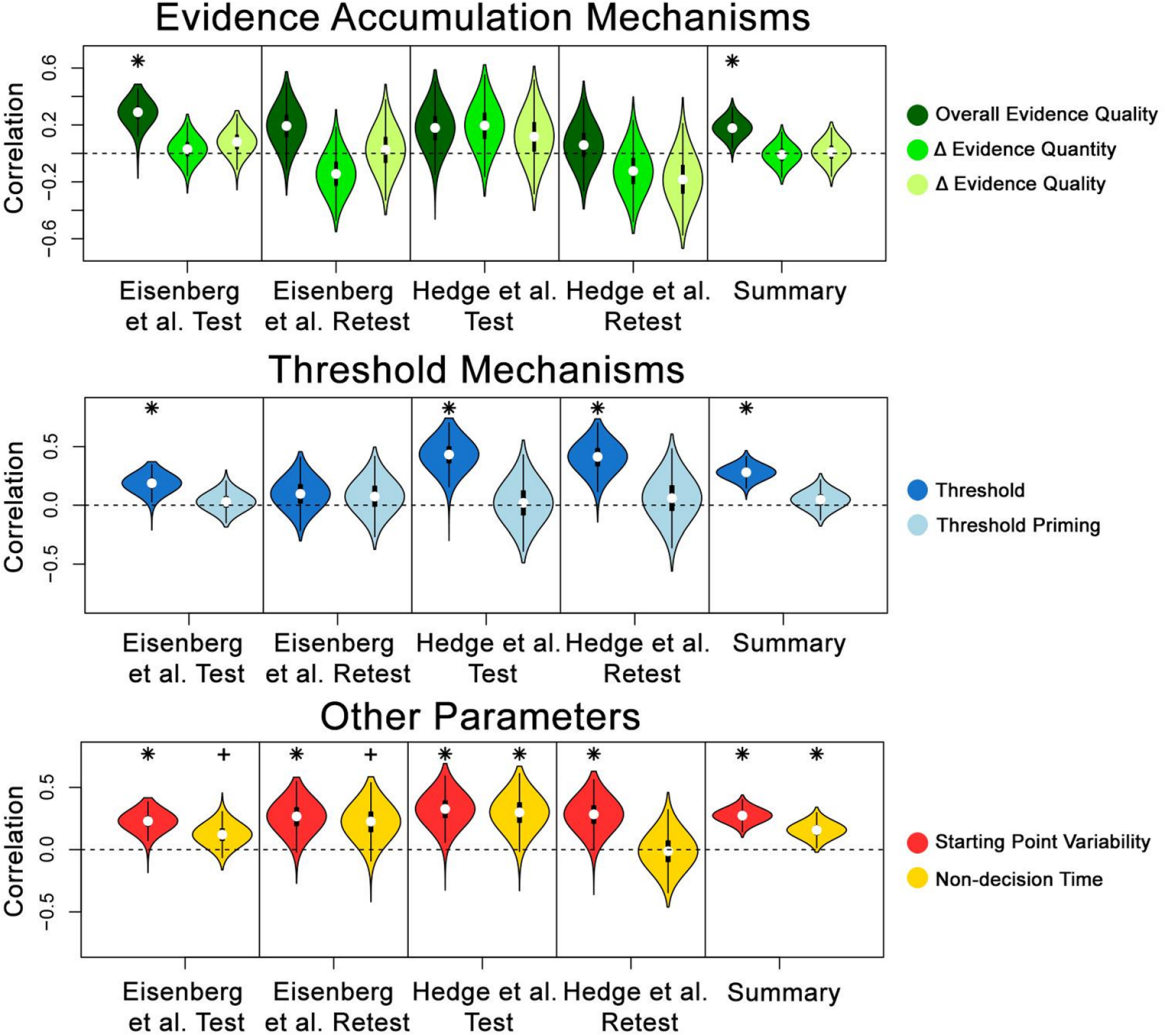
Posterior distributions of parameter correlations across conflict tasks.* indicates the 95% credible interval does not contain 0, and + indicates the 89% credible interval does not contain 0

After establishing reliability of the conflict LBA parameters within conflict tasks, we investigated which components of the model show evidence of task generality by exploring the covariance structure across different conflict tasks. The posteriors of the cross-task correlations are pictured in Fig. 4, and summary statistics for these posteriors can be found in Supplementary Tables S3 and S4. Here, the plots are broken up by evidence accumulation related parameters, threshold parameters, and starting point variability/non-decision time to compare conflict specific mechanisms (namely congruency effects on drift rate in row 1, and priming effects on threshold in row 2) across datasets.

There are three parameters that correspond to evidence accumulation: overall evidence quality, conflict effects on evidence quality, and conflict effects on evidence quantity. Overall, the correlation for overall evidence quality was higher (meta-analysis posterior mean = 0.18, see final row of Fig. 4 and Table S4) than the two conflict effects on evidence quality (0.01) and quantity (−0.01). Additionally, across the datasets, overall evidence quality is the only parameter that showed evidence for a consistently positive correlation on average, and for which the majority of posterior samples were above 0 across all task pairs. In contrast, posteriors for cross-task relations for the conflict effects have variable signs and are generally centered close to 0. Although the posterior for cross-task relations in overall evidence quality was only credibly different from 0 (95% CI) in the Eisenberg test dataset (which includes the Simon and Stroop task), this is likely due to increased estimation precision at a greater sample size, as the Eisenberg test sample includes 521 individuals, and the others have between 103 and 151 individuals.

Next, there are two parameters related to decision threshold: the overall threshold parameter assumed in the standard LBA, and the conflict LBA-specific parameter that adds a priming effect on threshold to explain transient effects of conflicting information on early evidence accumulation. Similarly to the evidence accumulation parameters, the overall threshold parameter had a higher cross task correlation than the threshold priming parameter (meta-analytic posterior means of 0.28 and 0.05 respectively), and for ¾ datasets the CI for the threshold parameter did not contain 0. The exception to this is in the Eisenberg retest (again including Simon and Stroop tasks) dataset, where the two posteriors are highly similar. In all four datasets, the priming parameter is nearly centered around 0, suggesting this priming parameter shows little evidence of task-generality across the Stroop, Flanker, and Simon tasks.

Finally, there are two remaining parameters of the LBA that are not changed in the conflict version of the model: starting point variability, and non-decision time. In all four datasets, the starting point variability CIs did not contain 0, and the mean correlation ranged from 0.23 to 0.32. There was less consistent evidence for task-generality in non-decision time. The 95% CI for the Hedge test dataset (which includes the Flanker and Stroop tasks), and the 89% CIs for the Eisenberg test and retest datasets did not contain 0. Interestingly though, in the Hedge retest dataset (containing the same tasks and individuals as the Hedge test dataset), the posterior for non-decision time was centered on 0.

### Cross-task Correlations between Conflict and Perceptual Decision-making Mechanisms

Across the different conflict tasks we found that the parameters with relatively high correlations were largely the non-conflict specific components, i.e., the parameters in the standard LBA (and evidence accumulation models more generally) that drive behavior across both conflict conditions. Our next goal, then was to look at the covariances between conflict tasks and a more basic perceptual decision task that is not assumed to have cognitive control demands, the numerosity discrimination task. Behavior in this task is captured well by evidence accumulation models, but it does not contain a specific conflict manipulation like the Stroop, Simon, and Flanker tasks. To explore this question, we used a third dataset, where participants completed either a Numerosity task and either a Simon or a Flanker task.

**Fig. 5:**
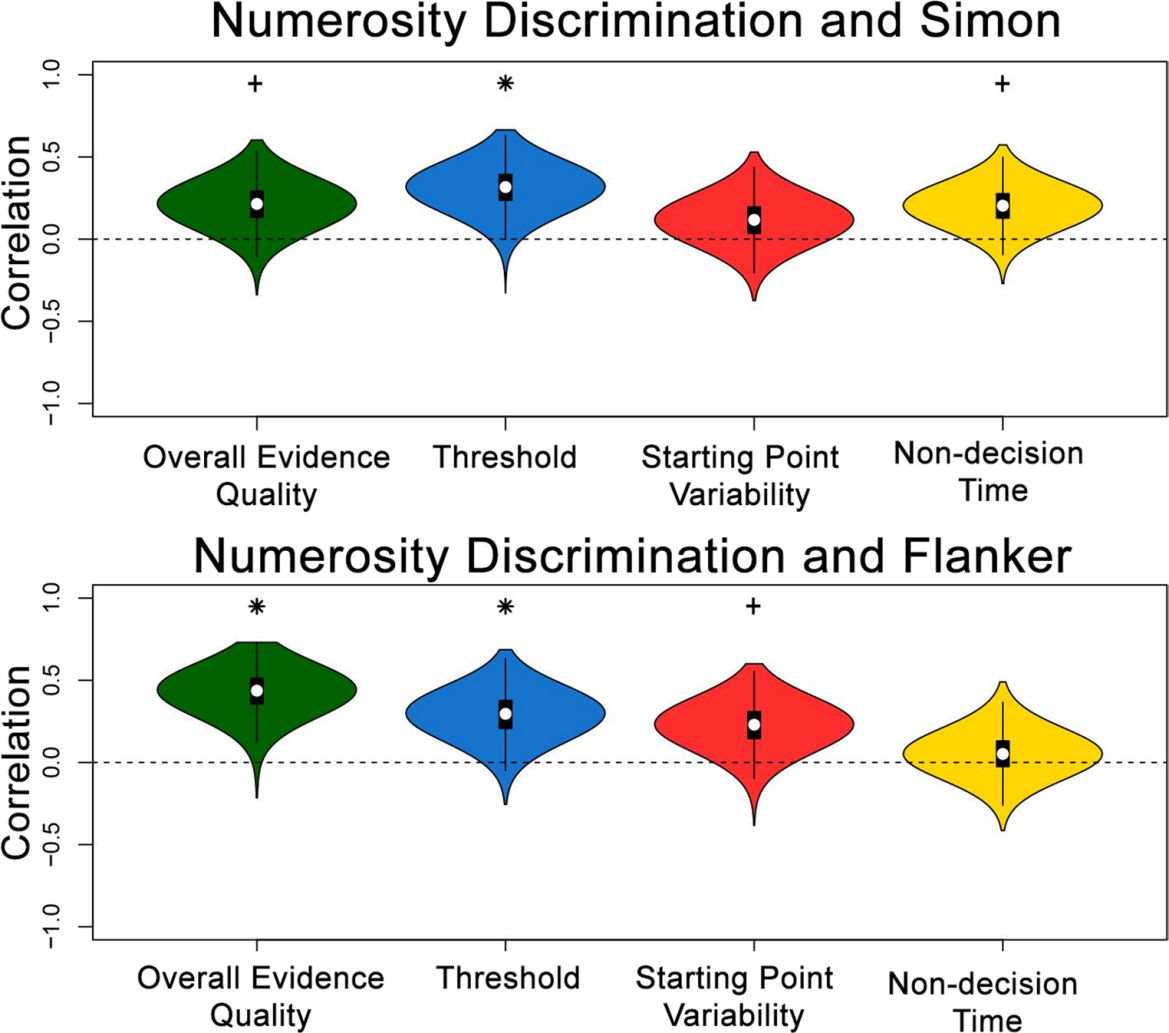
Posterior distributions of parameter correlations between conflict and numerosity tasks. * indicates the 95% credible interval does not contain 0, and + indicates the 89% credible interval does not contain 0

Because there is no priming or congruence mechanism in the numerosity task, only four parameters will be compared in the joint model (i.e., excluding evidence quantity, Δ evidence quality, and threshold priming). The resulting posterior distributions are shown in Fig. 5 and summarized in Supplementary Table S5. First, looking at the covariance structure between the numerosity and Simon task, all parameters had a mean positive correlation, where the 95% CI for threshold and the 89% CIs for overall evidence quality and non-decision time did not contain 0. Second, for the Flanker task and numerosity, again all parameters had a mean correlation above 0, although the posterior for non-decision time is close to 0. Overall evidence quality and threshold had 95% CIs above 0, while starting point variability had an 89% CI above 0. Comparing both tasks and numerosity to the results we observed across conflict tasks, it appears that threshold, and to a lesser degree, overall evidence quality, are stable sources of individual differences in behavior across tasks. A key difference between the conflict task covariances and the conflict-numerosity task covariances lies in starting point variability. In all four datasets for the conflict task, the vast majority (and all CIs) of the posteriors were above zero. This was not the case for the numerosity-conflict task, where the posterior means were lower, and larger amounts of the posteriors were below 0.

## Discussion

Previous research proposed two categories of mechanisms that potentially contribute to goal-directed responding on conflict tasks: (1) task-general mechanisms that contribute performance equally across the congruent and incongruent conditions of conflict tasks (indexed by efficiency of evidence accumulation, EEA), and (2) conflict-specific mechanisms selectively or preferentially engaged in the incongruent condition (often dubbed “attentional control mechanisms”). Here, we investigated trait-like contributions of these mechanisms, as well as contributions of other parameters (e.g., response threshold) to conflict task performance with hierarchical Bayesian joint models using the conflict LBA. The joint modeling approach maximizes precision of individuals’ parameter estimates and addresses attenuation of correlations due to measurement error, providing a powerful framework for identifying common mechanisms underlying individual differences in conflict task performance. We found evidence of moderate test-retest reliability within conflict tasks for parameters reflecting both conflict-specific processing mechanisms and task-general mechanisms, though task-general mechanisms exhibited higher reliability than conflict-specific mechanisms within individual datasets. However, we found no evidence of a shared conflict suppression mechanism that drives individual differences in performance across conflict tasks. Rather, task general mechanisms indexed by EEA showed evidence of generality across different conflict tasks and between conflict tasks and a simple decision-making task (i.e., numerosity discrimination) that is not traditionally assumed to involve conflict effects. These results further confirm that EEA indexes a trait-like ability to select goal-relevant information from the environment that contributes generally to performance across tasks and conditions irrespective of whether they involve conflict. In contrast, conflict suppression effects appear to be idiosyncratic to specific tasks rather than reflecting a general ability for conflict suppression or “attentional control”. Consistent with the assumptions of the conflict LBA model, we found that behavior in conflict tasks across datasets was best explained by including two conflict-specific mechanisms that account for (1) the early transient influence of conflict on processing, implemented by “priming” effects in the threshold parameter, (2) the persistent influence of conflict on processing throughout the trial implemented by drift rate parameters. These findings are consistent with prior work using the conflict LBA in other samples (Weigard et al., 2019; Weigard, Huang-Pollock, Brown et al., 2018a; Weigard, Huang-Pollock, Heathcote et al., 2018b) and supports the general notion, first proposed in conflict DDMs, that the influence of conflicting information impacts evidence accumulation, and its impact is especially prominent early in the decision process.

We additionally found that within conflict tasks, most parameters displayed some evidence of modest test-retest reliability, including both general decision-making and conflict-specific parameters. However, there were large differences across parameters, where meta-analytic correlations ranged from an average of 0.21 for threshold priming to 0.58 for starting point variability. The test-retest reliability of the Hedge dataset can be directly compared to the test-retest reliability reported in Rouder and Haaf (2019) who analyzed the same dataset in a hierarchical Bayesian framework using the difference score approach applied to behavioral summary statistics (i.e., RT). Rouder and Haaf found a test-retest reliability of the Stroop and Flanker tasks to be 0.72 and 0.68 compared to our ranges of 0.2 to 0.74 and 0.11 to 0.59 for the Stroop and Flanker tasks. The highest reliabilities were found in starting point variability, but not parameters related to conflict.

It is likely that our estimates are lower than those of Rouder and Haaf (2019) because we focus on dissociable mechanisms of performance, whereas summary statistics like RT lump several of these mechanisms together. Therefore, our results point to a trade-off between the specificity of model-based performance measures and their reliability. Composite measures such as RT may provide more reliable estimates, but their multidetermined nature nonetheless limits their validity as they collapse several mechanisms into a single measure. Cognitive model parameters that are less psychologically ambiguous may therefore be preferable to composite measures, even if their reliability is more modest. As noted by Oberauer (2024), efforts to enhance reliability at the cost of specificity can be counterproductive to the goal of measuring attentional control mechanisms.

We found that only the general decision-making parameters core to evidence accumulation models, but not the added conflict-task-specific parameters, were correlated across different conflict tasks. This result corroborates the conclusions of Hedge et al. (2022) by generalizing their findings to a different model and different datasets. In addition, it provides a critical extension of their work by using a Bayesian framework to demonstrate that conflict related parameters are not correlated across tasks, even when accounting for measurement noise and attenuation. Notably, across the two studies, analogous model mechanisms were the most consistent across tasks, with Hedge et al. finding the highest cross-task correlations in the drift rate and boundary separation parameters, where we found the highest cross-task correlations in overall evidence quality and threshold.

The median posterior estimates reported here for cross-task correlations were slightly lower than the point estimates reported by Hedge et al., which may be due in part to accounting for measurement error in our hierarchical Bayesian framework. Another important consideration relevant to measurement error in this context is error related to imperfect recovery of model parameters (White et al., 2018). Although we found that the conflict LBA’s parameter recovery was generally good, it was less than perfect, with an average correlation of approximately *r* = 0.80 in the Eisenberg et al. test dataset (Supplementary Figures S10-11). Hence, it is possible that this less-than-perfect recovery contributed to the attenuation of test-retest reliability estimates or cross-task correlations. However, it is unlikely to fully explain our findings of a complete lack of evidence for cross-task correlations in conflict task-specific parameters.

In addition, the effect sizes observed for cross-task correlations in evidence quality/EEA were highly statistically significant but relatively modest in size (0.18 for meta-analytic correlations across conflict tasks, 0.21 and 0.43 for correlations between conflict tasks and the numerosity task). We attribute this modest effect size to two factors. The first is absence of latent factor modeling in the present study, which helps separate EEA-related variance shared across disparate tasks from idiosyncratic factors that contribute to performance on individual tasks. In our prior work in the Eisenberg sample (Weigard et al., 2021), which did employ latent factor modeling (but did not use Bayesian joint modeling), we observed very strong loadings of drift rates from individual task conditions onto a shared latent factor (mean *β* = 0.55; range = 0.41–0.69) despite more modest manifest correlations among these drift rates (median *r* = 0.29; range = 0.14–0.43). Future work that uses joint modeling in conjunction with latent factor modeling of EEA and other evidence accumulation model parameters (e.g., Stevenson et al., 2024) would provide a better idea of how strongly measures from individual tasks cohere with such a general factor.

Second, although our Bayesian joint modeling framework can partially account for attenuation of cross-task correlations due to measurement error, there is a second kind of attenuation that arises from test-retest reliability of the individual tasks that remains unaddressed. Applying Spearman’s correction for this type of attenuation (Trafimow, 2016) and assuming a test-retest reliability of 0.44 (i.e., the observed reliability for evidence quality/EEA parameters) to the meta-analytic correlation of 0.18, gives $\:0.18/\sqrt{0.44*0.44}\:$= 0.41. Therefore, the observed cross-task correlations in evidence quality/EEA parameters are likely modest in part due to the fair test-retest reliability of these parameters in individual tasks. Again, the use of latent factors offers an important future direction given that these factors tend to have better test-retest reliability (Weigard et al., 2021) than measures from individual tasks (Enkavi et al., 2019).

Finally, these general decision-making parameters were correlated between conflict tasks and a numerosity task, a simple decision-making task without an explicit conflict manipulation. These results are in line with two prior studies that used point estimates from the standard DDM in structural equation modeling frameworks to identify general factors for efficiency of evidence accumulation on tasks thought to measure higher order “executive” or “control” functions (Löffler et al., 2024; Weigard et al., 2021). Both studies found that simple decision tasks that are not traditionally thought to measure such functions nonetheless displayed strong relations with the general factor, suggesting that such a factor has little to do with specific higher-order “executive” or “control” processes and instead reflects individuals’ general efficiency of evidence accumulation (EEA) that drives performance across many tasks. Our findings provide strong evidence for the importance of this cross-task factor by documenting relations across conflict and non-conflict tasks using a formal modeling framework that can flexibly explain mechanisms of performance in both types of tasks (i.e., LBA) and optimal methods for quantifying cross-task correlations while accounting for measurement error.

These findings are notable in the context of current debates about the construct validity and measurement of “attention control” (Burgoyne et al., 2023; Mashburn et al., 2024; Oberauer, 2024; von Bastian et al., 2020), which is posited to be a general factor that allows people to attend to task-relevant information, suppress attention to task-irrelevant information, and control their behavior more broadly. This construct has often been assumed to be measured with difference scores between congruent and incongruent conditions of conflict tasks, as well as more recently through parameters from conflict DDMs, although these measurement assumptions are limited by a general lack of clarity in attentional control’s definition (for an overview of these limitations, see: Oberauer, 2024). Our findings provide compelling evidence against the notion that conflict suppression mechanisms reflect a person’s general ability to control attention, given that these mechanisms appear to be idiosyncratic across different conflict tasks.

### Defining Attentional Control

The question of whether the task-general processes we did identify, including individuals’ EEA, can instead be thought of as indices of “attentional control” is complex. Our measure of EEA from the LBA is the difference between drift rates of accumulators matching the correct response and those mismatching the correct response. This reflects a person’s (apparently task-general) ability to selectively accumulate task-relevant information rather than task-irrelevant information to inform decisions, which certainly has some conceptual similarity to earlier definitions of attentional control (Weigard et al., 2021). However, as individual differences in the quality of evidence accumulation appear to be ubiquitous, even across tasks with no explicit demands on attentional functioning, such a definition of attentional control could be difficult to separate from other factors that may have a general impact on performance.

Oberauer’s (2024) recent review on conceptual and methodological challenges to defining attentional control directly addressed this challenge by conducting simulations assuming that variance in performance in the incongruent (higher demands on attentional control) condition, Ψ_1_, and the congruent (lower demands on attentional control) condition, Ψ_0_, is determined by several factors. These include systematic influences unrelated to the construct of interest (*X*), attentional control (*AC*) and its differential influence on the incongruent, α_1_, and congruent, α_0_, condition, and normally distributed noise, σ^2^:

$$\:{{\Psi\:}}_{1}=X+\:{\alpha\:}_{1}AC+\:{\sigma\:}^{2}$$

$$\:{{\Psi\:}}_{0}=X+\:{\alpha\:}_{0}AC+\:{\sigma\:}^{2}$$

When the influence of *AC* on the incongruent condition (α_1_) is substantially larger than the influence of AC on the congruent condition (i.e., α_1_ > α_0_), simulations demonstrate that difference scores become valid measures of attentional control. When α_1_ and α_0_ are equivalent, difference scores have no validity for measuring attentional control. In this situation, measures that aggregate performance metrics across both the congruent and incongruent conditions (e.g., the latent factors in: Weigard et al., 2021) do have validity for measuring attentional control, but are also problematic because they are contaminated by unwanted variance related to other systematic sources (*X*). The models fit in the current study explicitly assume that EEA has an equivalent influence across task conditions, but that conflict-specific control (CSC) has a differential influence, which is reflected in the following modifications to Oberauer’s equations:

$$\:{{\Psi\:}}_{1}=X+EEA+\:{\alpha\:}_{1}CSC+\:{\sigma\:}^{2}$$

$$\:{{\Psi\:}}_{0}=X+EEA+\:{\alpha\:}_{0}CSC+\:{\sigma\:}^{2}$$

These equations make clear that difference scores in manifest performance measures (Ψ_1_ - Ψ_0_), such as RT and accuracy, may be valid measures of CSC under some conditions, but will completely fail to measure EEA. If EEA reflects a task-general ability to selectively accumulate goal-relevant information and if this ability is relevant to self-control more broadly, it would be expected that difference scores in manifest performance measures would be unrelated to relevant criterion measures (e.g., questionnaire measures of self-control, clinical phenotypes that involve self-control difficulties) but that measures of performance that aggregate across congruent and incongruent conditions would instead show validity in predicting these criterion measures. This pattern of findings is indeed what is reflected in the literature (Weigard et al., 2021; Weigard & Sripada, 2021).

The equations demonstrate that manifest measures (Ψ_1_, Ψ_0_) will continue to be polluted by other systematic sources of variance (*X*). Using a cognitive model-based framework provides some amelioration of this problem because it allows for explicit estimation of EEA separately from other mechanistic parameters that would be contained in *X*, including individuals’ response thresholds and non-decision times. However, a limitation of using cognitive modeling alone is that the observed EEA on a given task is likely to reflect not only individuals’ task-general ability to selectively gather goal-relevant information but is also likely to reflect individuals’ perceptual discrimination ability within that task’s specific domain (e.g., numerosity, language, spatial processing). Latent factor models can be used to explicitly estimate a task-general factor that influences observed EEA across many domains and to parse this factor from domain- and task-specific abilities. Indeed, latent factor modeling applications to DDM parameters have suggested that drift rate shows both domain-specific factors and a domain-general factor (Lerche et al., 2020), the latter of which could be analogous to the task-general EEA factor posited in this work. Hence, the combination of cognitive modeling with cross-task factor modeling in diverse task batteries, ideally within a joint modeling framework that simultaneously estimates parameters of each (e.g., Stevenson et al., 2024), offers a promising way forward for measuring individuals’ task-general ability to selectively gather goal-relevant information. To the extent that this ability could be characterized as a form of task-general “attentional control” or “cognitive control”, such work could provide a critical advance to the measurement of such constructs.

### Limitations

The current study has several limitations. First, we investigated the most commonly studied conflict tasks, but numerous other tasks exist, such as the antisaccade task (Hallett, 1978). However, recent modeling work suggests that the antisaccade task primarily captures individual differences in processing speed, rather than in conflict processing or cognitive control (Frischkorn & Oberauer, 2025; Rouder et al., 2022). This echoes the predominance of general task processes observed both in the current study and in previous work on other conflict tasks (Hedge et al., 2022; Weigard et al., 2021). Future research could investigate whether the mode of responding (e.g., saccade, button-press, or verbal response) affects individual differences in decision making under conflict. Second, we chose to focus on standard versions of the Flanker, Stroop, and Simon tasks. However, more complex versions of these tasks have also been proposed, such as the Emotional Stroop task (Williams et al., 1996), which can modify how people perform these tasks and often recruit different or additional cognitive processes. Additionally, when comparing cross-conflict task differences, we investigated Stroop versus Simon or Flanker tasks, but did not directly investigate Simon versus Flanker within a joint model, as the datasets utilized here did not have the same individuals complete both tasks. We observed differences in the Simon and Flanker tasks in terms of test-retest reliability and correlations to the Numerosity task, but further work is needed to provide a complete picture of the differences between different conflict tasks. Previous work has suggested different conflict tasks utilize different suppression processes (Egner, 2008), so conflict tasks that recruit the same processes could show correlations between conflict parameters.

Third, the individuals examined here had low error rates (the highest average error rate across all tasks and datasets was approximately 10%), which can reduce parameter identifiability and the ability to distinguish between different model variants (Lüken et al., 2025). While we ameliorated this limitation by fixing v, future work can increase the difficulty of the task to improve identifiability, by e.g., requiring faster responses or decreasing stimulus discriminability, though it is unclear if these manipulations would also affect task strategy. Finally, we focused on correlations of individual cognitive model parameters across tasks (e.g., drift rate on the Flanker with drift rate on the Stroop), but did not investigate the possibility that several of these individual parameters could be driven by common factors (e.g., a factor that explains variance in both drift rates and response thresholds across both tasks). Additionally, while the conflict parameters exhibited lower test-retest reliability separately, a common “conflict” factor could yield higher reliability. Although doing so was beyond the scope of the hypotheses tested in the current study, future work could apply exploratory factor analysis within a joint modeling framework (Stevenson et al., 2024) to assess this possibility.

## Conclusions

In sum, this is the first study to simultaneously leverage the advantages of formal cognitive process models and hierarchical Bayesian joint modeling methods to assess the test-retest reliability and cross-task generalizability of mechanisms that drive individuals’ performance in common conflict suppression paradigms, including the Flanker, Stroop, and Simon tasks. We find that all these mechanisms display some degree of test-retest reliability, including both general mechanisms of decision-making, such as evidence accumulation and response caution, and mechanisms specific to conflict processing. However, only the general mechanisms of decision-making appear to drive individual differences in performance across conflict tasks, as well as across a simpler decision-making task with no explicit demands on conflict suppression. Findings support the robustness of individual differences in general computational mechanisms of decision-making while indicating that the specific mechanisms measured in conflict paradigms are idiosyncratic to tasks rather than general mechanisms of behavior.

## Supplementary Information

Below is the link to the electronic supplementary material.

Supplementary Material 1

## Data Availability

As described in their original publications, the Hedge et al. (2018) dataset is available on the Open Science Framework (https://osf.io/cwzds/), and the Eisenberg et al. (2019) dataset is available on GitHub (https://github.com/IanEisenberg/Self_Regulation_Ontology/tree/master/Data/Complete_02-16-2019).
