# Supplementary material for: Joint Cognitive Models Reveal Sources of Robust Individual Differences in Conflict Processing
Source: Comput Brain Behav. 2026 Mar 19;9(2):319–36. doi: 10.1007/s42113-026-00263-1 (PMC13004055; doi:10.1007/s42113-026-00263-1)
Supplement: Supplementary file 1 — Supplementary Material 1 [file 42113_2026_263_MOESM1_ESM.docx]

**Supplementary Tables**

**Table S1**

*Summary statistics for posterior distributions of parameter correlations within conflict tasks*

| Dataset | Task | Mechanism | Mean | 95% CI (lower) | 95% CI (upper) | Standard deviation |
| --- | --- | --- | --- | --- | --- | --- |
| Eisenberg | Stroop | Overall Evidence Quality | 0.55 | 0.37 | 0.71 | 0.1 |
|  |  | Δ Evidence Quantity | 0.47 | 0.25 | 0.64 | 0.1 |
|  |  | Δ Evidence Quality | 0.43 | 0.08 | 0.63 | 0.13 |
|  |  | Threshold | 0.5 | 0.32 | 0.64 | 0.09 |
|  |  | Threshold Priming | 0.27 | 0.05 | 0.49 | 0.11 |
|  |  | Starting Point Variability | 0.53 | 0.35 | 0.68 | 0.09 |
|  |  | Non-decision Time | 0.31 | 0.09 | 0.5 | 0.11 |
|  | Simon | Overall Evidence Quality | 0.31 | 0.07 | 0.52 | 0.12 |
|  |  | Δ Evidence Quantity | 0.13 | -0.13 | 0.37 | 0.13 |
|  |  | Δ Evidence Quality | 0.15 | -0.12 | 0.41 | 0.13 |
|  |  | Threshold | 0.23 | 0 | 0.45 | 0.12 |
|  |  | Threshold Priming | 0.23 | -0.01 | 0.45 | 0.12 |
|  |  | Starting Point Variability | 0.47 | 0.27 | 0.63 | 0.1 |
|  |  | Non-decision Time | 0.24 | 0.02 | 0.46 | 0.11 |
| Hedge | Stroop | Overall Evidence Quality | 0.53 | 0.31 | 0.7 | 0.1 |
|  |  | Δ Evidence Quantity | 0.42 | 0.15 | 0.63 | 0.12 |
|  |  | Δ Evidence Quality | 0.3 | -0.01 | 0.56 | 0.15 |
|  |  | Threshold | 0.46 | 0.26 | 0.63 | 0.1 |
|  |  | Threshold Priming | 0.2 | -0.08 | 0.46 | 0.14 |
|  |  | Starting Point Variability | 0.74 | 0.62 | 0.83 | 0.07 |
|  |  | Non-decision Time | 0.22 | -0.02 | 0.45 | 0.12 |
|  | Flanker | Overall Evidence Quality | 0.36 | 0.14 | 0.56 | 0.11 |
|  |  | Δ Evidence Quantity | 0.37 | 0.12 | 0.59 | 0.12 |
|  |  | Δ Evidence Quality | 0.48 | 0.24 | 0.66 | 0.11 |
|  |  | Threshold | 0.53 | 0.32 | 0.72 | 0.11 |
|  |  | Threshold Priming | 0.11 | -0.29 | 0.59 | 0.22 |
|  |  | Starting Point Variability | 0.59 | 0.42 | 0.73 | 0.08 |
|  |  | Non-decision Time | 0.58 | 0.39 | 0.73 | 0.1 |

*Note: 95% CI = 95% credible interval*

**Table S2**

*Meta-analysis summary statistics for posterior distributions of parameter correlations within conflict tasks*

| Mechanism | Mean | 95% CI (lower) | 95% CI (upper) | Standard deviation |
| --- | --- | --- | --- | --- |
| Overall Evidence Quality | 0.44 | 0.33 | 0.53 | 0.05 |
| Δ Evidence Quantity | 0.35 | 0.22 | 0.46 | 0.06 |
| Δ Evidence Quality | 0.34 | 0.21 | 0.46 | 0.07 |
| Threshold | 0.43 | 0.33 | 0.52 | 0.05 |
| Threshold Priming | 0.21 | 0.06 | 0.36 | 0.08 |
| Starting Point Variability | 0.58 | 0.49 | 0.66 | 0.04 |
| Non-decision Time | 0.34 | 0.23 | 0.44 | 0.05 |

*Note: 95% CI = 95% credible interval*

**Table S3**

*Summary statistics for posterior distributions of parameter correlations across conflict tasks*

| Dataset | Task | Mechanism | Mean | 95% CI (lower) | 95% CI (upper) | Standard deviation |
| --- | --- | --- | --- | --- | --- | --- |
| Eisenberg | Stroop- Simon Test | Overall Evidence Quality | 0.28 | 0.11 | 0.4 | 0.07 |
|  |  | Δ Evidence Quantity | 0.03 | -0.11 | 0.17 | 0.07 |
|  |  | Δ Evidence Quality | 0.08 | -0.06 | 0.21 | 0.07 |
|  |  | Threshold | 0.19 | 0.05 | 0.3 | 0.06 |
|  |  | Threshold Priming | 0.03 | -0.1 | 0.16 | 0.07 |
|  |  | Starting Point Variability | 0.23 | 0.09 | 0.34 | 0.06 |
|  |  | Non-decision Time | 0.12 | -0.02 | 0.25 | 0.07 |
|  | Stroop- Simon Retest | Overall Evidence Quality | 0.19 | -0.04 | 0.41 | 0.12 |
|  |  | Δ Evidence Quantity | -0.14 | -0.36 | 0.1 | 0.12 |
|  |  | Δ Evidence Quality | 0.02 | -0.23 | 0.27 | 0.13 |
|  |  | Threshold | 0.1 | -0.13 | 0.31 | 0.11 |
|  |  | Threshold Priming | 0.07 | -0.17 | 0.31 | 0.12 |
|  |  | Starting Point Variability | 0.26 | 0.05 | 0.46 | 0.11 |
|  |  | Non-decision Time | 0.22 | -0.01 | 0.44 | 0.12 |
| Hedge | Stroop- Flanker Test | Overall Evidence Quality | 0.18 | -0.06 | 0.4 | 0.12 |
|  |  | Δ Evidence Quantity | 0.19 | -0.08 | 0.45 | 0.13 |
|  |  | Δ Evidence Quality | 0.11 | -0.18 | 0.39 | 0.14 |
|  |  | Threshold | 0.43 | 0.22 | 0.61 | 0.1 |
|  |  | Threshold Priming | 0.02 | -0.27 | 0.31 | 0.15 |
|  |  | Starting Point Variability | 0.32 | 0.12 | 0.5 | 0.1 |
|  |  | Non-decision Time | 0.29 | 0.05 | 0.51 | 0.12 |
|  | Stroop- Flanker Retest | Overall Evidence Quality | 0.06 | -0.18 | 0.29 | 0.12 |
|  |  | Δ Evidence Quantity | -0.12 | -0.37 | 0.14 | 0.13 |
|  |  | Δ Evidence Quality | -0.18 | -0.45 | 0.11 | 0.14 |
|  |  | Threshold | 0.41 | 0.17 | 0.6 | 0.11 |
|  |  | Threshold Priming | 0.06 | -0.25 | 0.35 | 0.16 |
|  |  | Starting Point Variability | 0.28 | 0.07 | 0.47 | 0.1 |
|  |  | Non-decision Time | -0.01 | -0.25 | 0.23 | 0.12 |

*Note: 95% CI = 95% credible interval*

**Table S4**

*Meta-analysis summary statistics for posterior distributions of parameter correlations across conflict tasks*

| Mechanism | Mean | 95% CI (lower) | 95% CI (upper) | Standard deviation |
| --- | --- | --- | --- | --- |
| Overall Evidence Quality | 0.18 | 0.06 | 0.28 | 0.05 |
| Δ Evidence Quantity | -0.01 | -0.12 | 0.1 | 0.06 |
| Δ Evidence Quality | 0.01 | -0.11 | 0.13 | 0.06 |
| Threshold | 0.28 | 0.18 | 0.37 | 0.05 |
| Threshold Priming | 0.05 | -0.08 | 0.17 | 0.06 |
| Starting Point Variability | 0.27 | 0.18 | 0.36 | 0.05 |
| Non-decision Time | 0.16 | 0.05 | 0.26 | 0.05 |

*Note: 95% CI = 95% credible interval*

**Table S5**

*Summary statistics for posterior distributions of parameter correlations between conflict and numerosity tasks*

| Tasks | Mechanism | Mean | 95% CI (lower) | 95% CI (upper) | Standard deviation |
| --- | --- | --- | --- | --- | --- |
| Flanker- Numerosity | Overall Evidence Quality | 0.43 | 0.18 | 0.63 | 0.12 |
|  | Threshold | 0.29 | 0.03 | 0.51 | 0.12 |
|  | Starting Point Variability | 0.22 | -0.04 | 0.45 | 0.12 |
|  | Non-decision Time | 0.05 | -0.18 | 0.3 | 0.12 |
| Simon- Numerosity | Overall Evidence Quality | 0.21 | -0.03 | 0.43 | 0.12 |
|  | Threshold | 0.31 | 0.07 | 0.53 | 0.12 |
|  | Starting Point Variability | 0.11 | -0.13 | 0.34 | 0.12 |
|  | Non-decision Time | 0.2 | -0.02 | 0.4 | 0.11 |

*Note: 95% CI = 95% credible interval*

**Supplementary Figures**


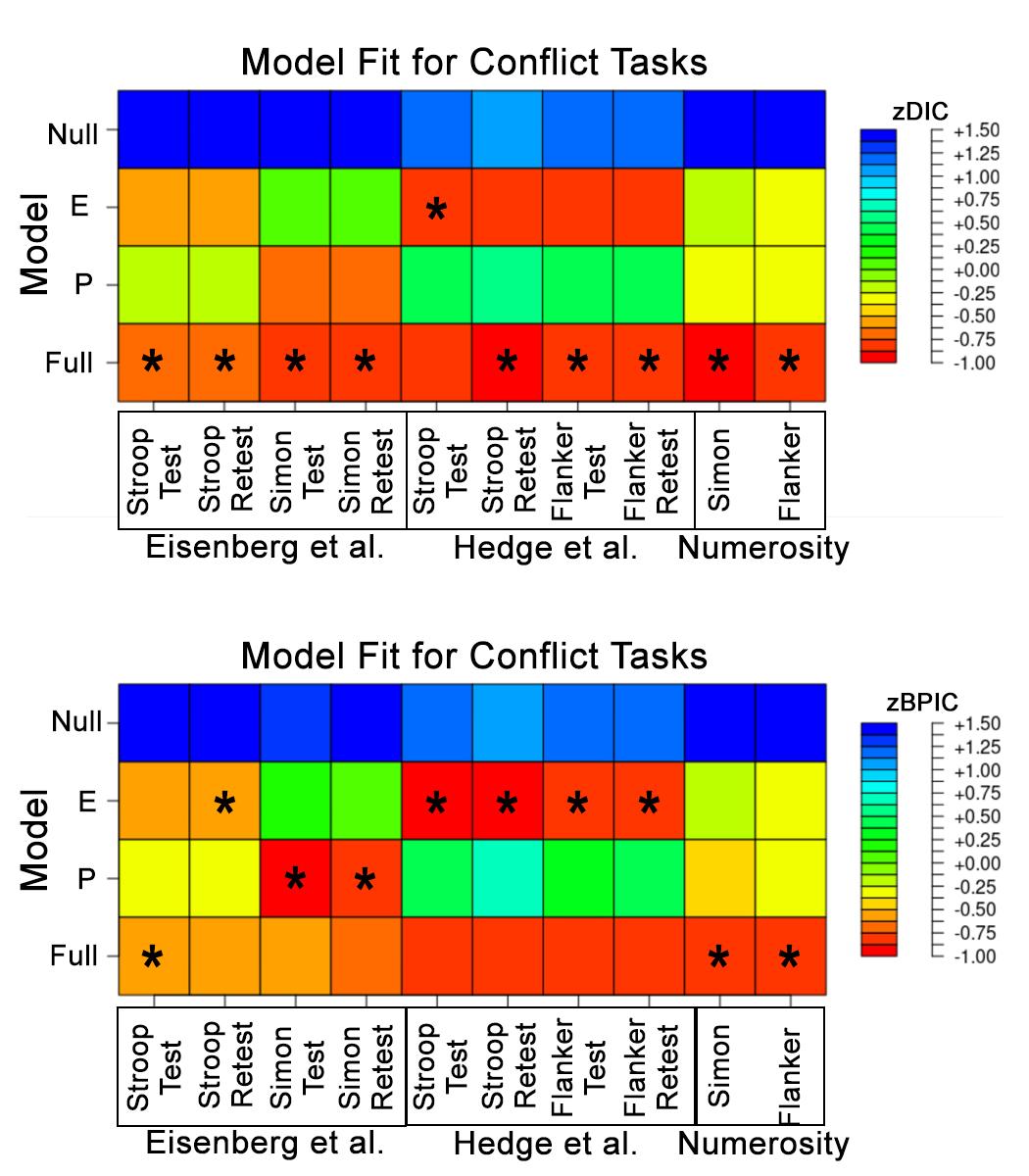


*Figure S1.* **Conflict Model Comparison using BPIC**. Asterisks indicate the winning model for each dataset/task. Model fit penalized by complexity is quantified here using the Bayesian predictive information criterion, or BPIC (Ando, 2007). The model with the lowest BPIC for each task (columns) is considered the winning model (indicated by asterisks). For plotting purposes, BPIC is z-scored within each column.


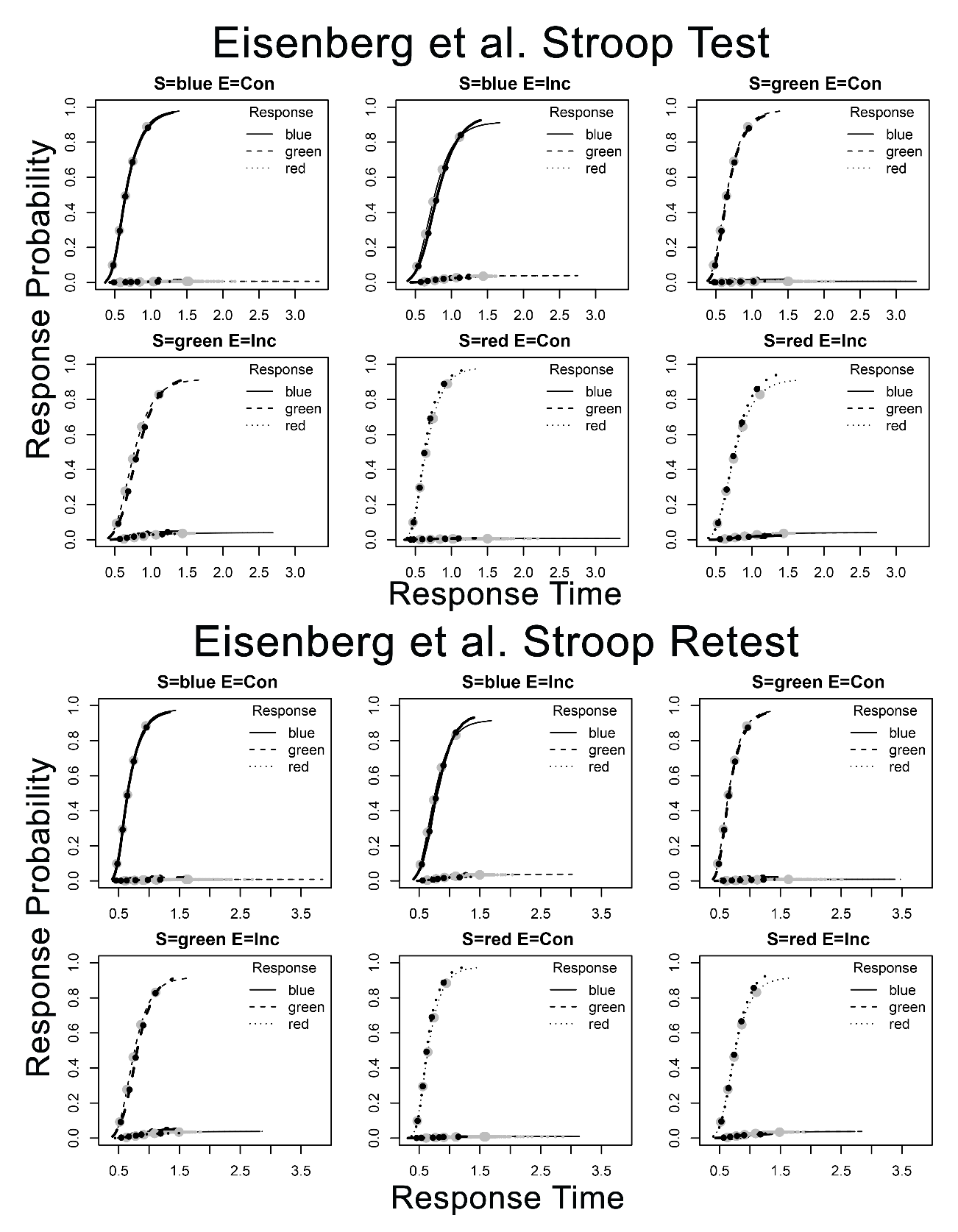


*Figure S2.* **Full Model Fits to Eisenberg et al. Stroop Task**. Gray points indicate model predictions and black points denote the real observed data. S denotes the correct response, and E indicates the condition where Inc=incongruent and Con=congruent.


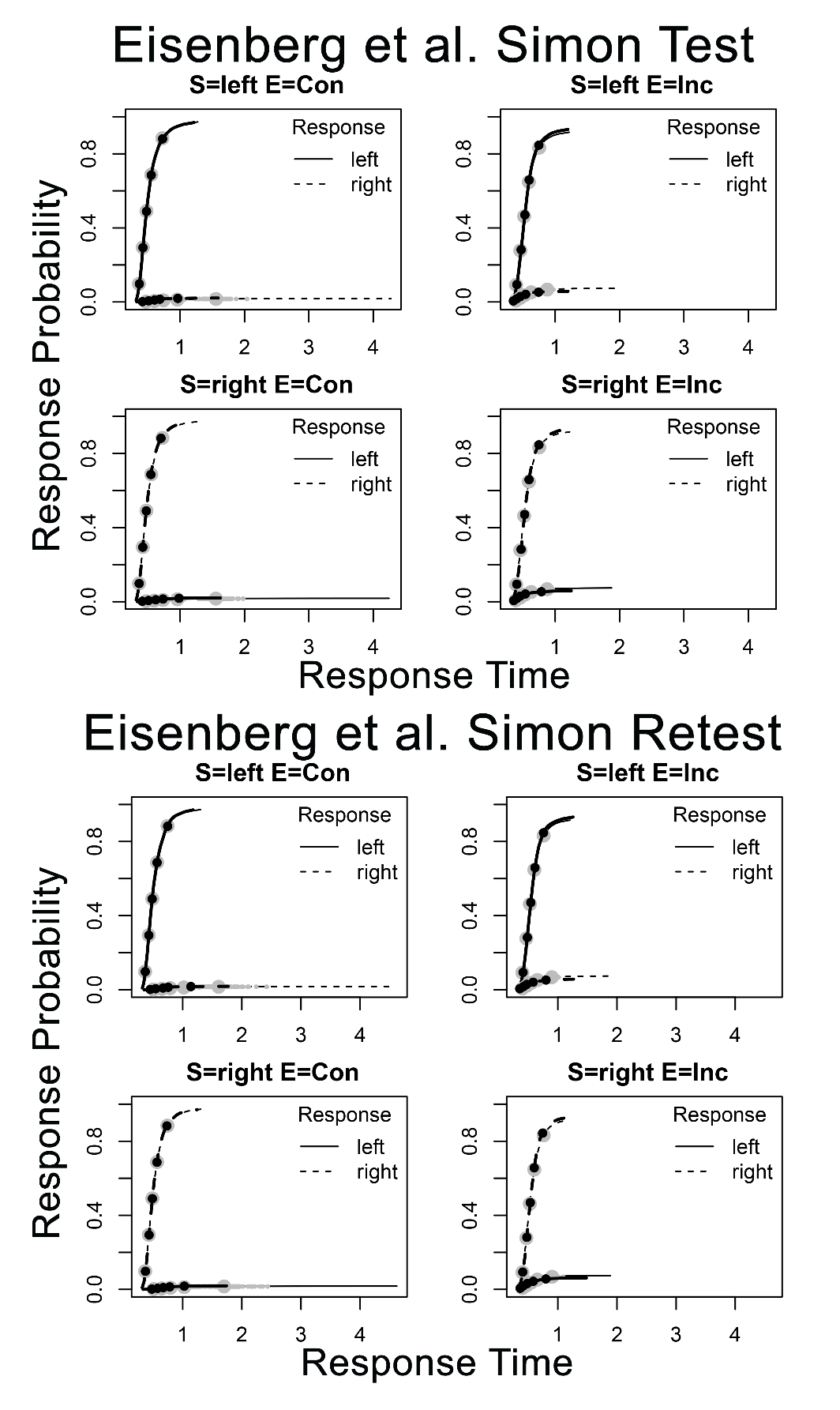


*Figure S3.* **Full Model Fits to Eisenberg et al. Simon Task**. Gray points indicate model predictions and black points denote the real observed data. S denotes the correct response, and E indicates the condition where Inc=incongruent and Con=congruent.

*
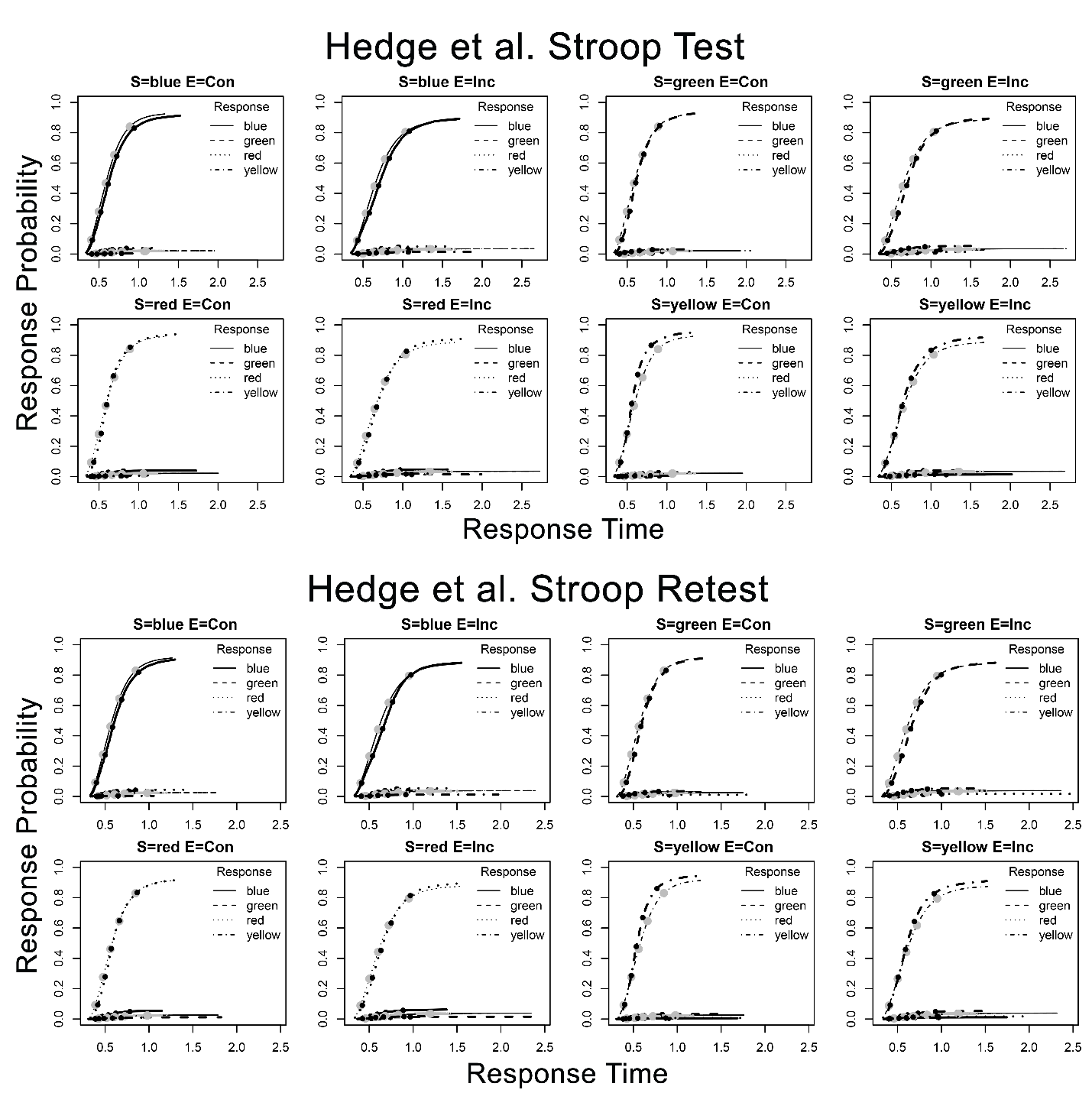
Figure S4.* **Full Model Fits to Hedge et al. Stroop Task**. Gray points indicate model predictions and black points denote the real observed data. S denotes the correct response, and E indicates the condition where Inc=incongruent and Con=congruent.


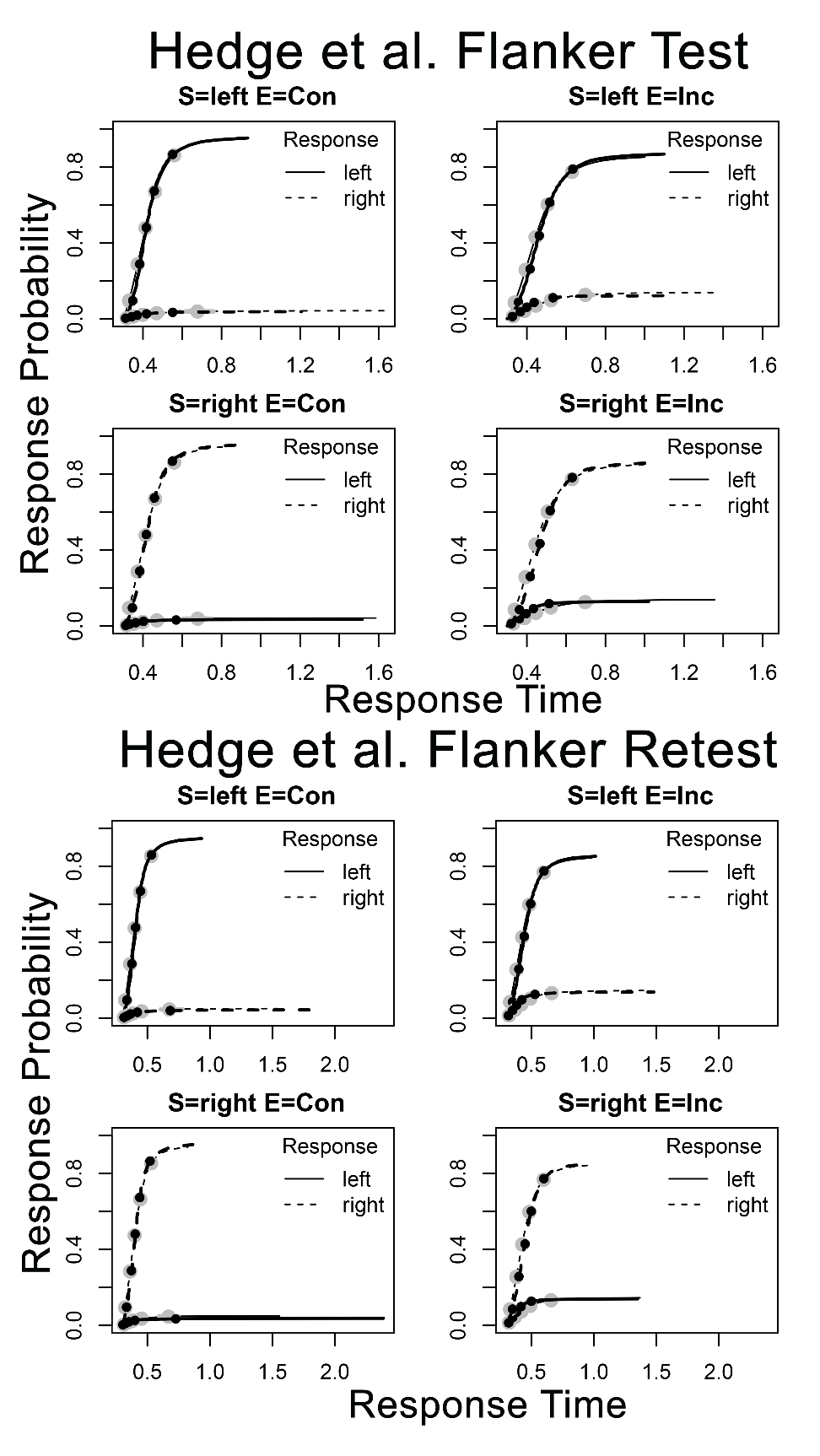


*Figure S5.* **Full Model Fits to Hedge et al. Flanker Task.** Gray points indicate model predictions and black points denote the real observed data. S denotes the correct response, and E indicates the condition where Inc=incongruent and Con=congruent.

*
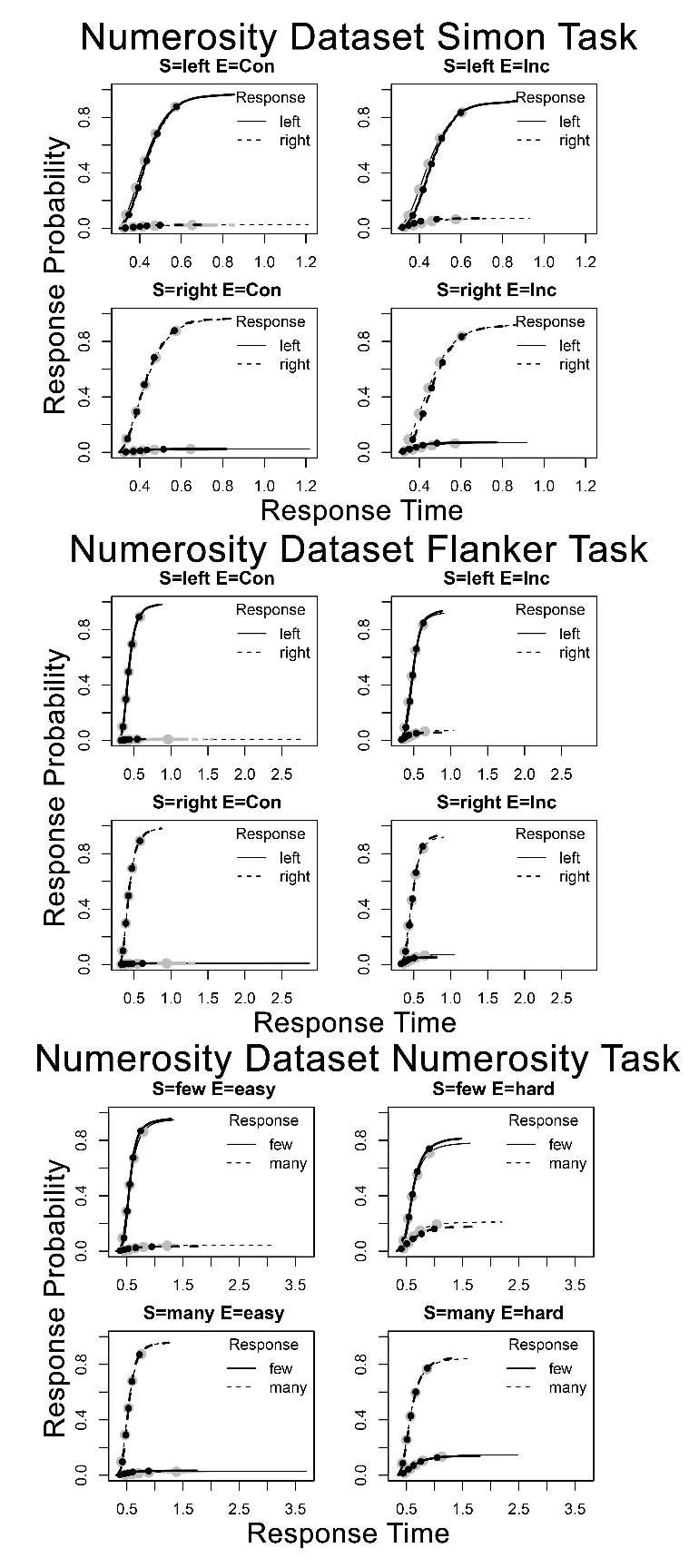
*

*Figure S6.* **Full Model Fits to Numerosity Dataset Simon, Flanker, and Numerosity Discrimination Tasks.** Gray points indicate model predictions and black points denote the real observed data. S denotes the correct response, and E indicates the condition where Inc=incongruent and Con=congruent.


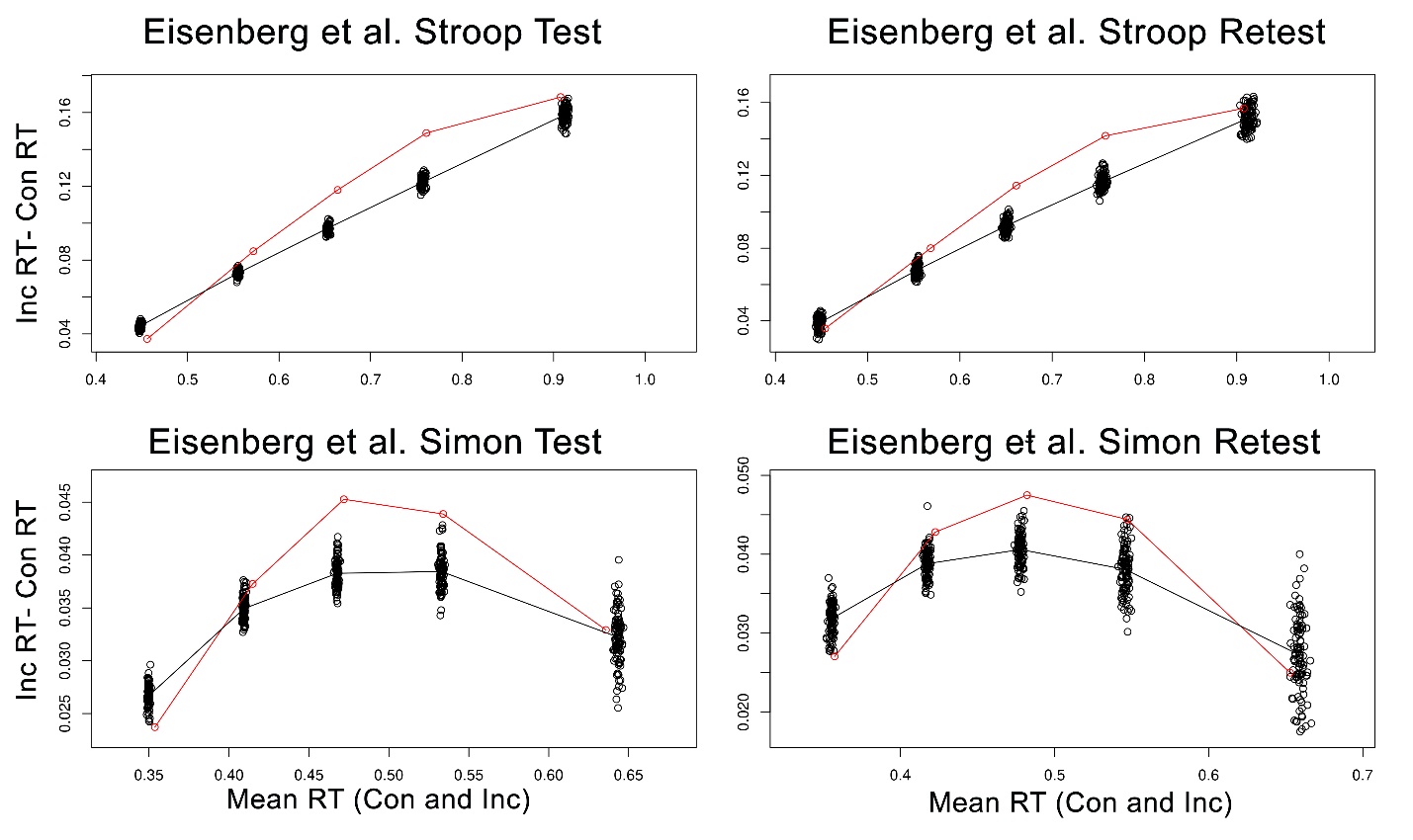


*Figure S7.* **Full Model Delta Function Fits for the Eisenberg et al. Dataset Stroop and Simon Tasks.** Group-level delta functions representing the difference between congruent (Con) and incongruent (Inc) correct RT quantiles (.1, .3, .5, .7, and .9) on the y-axis against the congruent/incongruent mean on the x-axis, for empirical data (red points and lines) and data predicted by 100 posterior samples from the model (black points indicate the data predicted by each individual sample; black lines indicate the average of data predicted by all posterior samples).

**
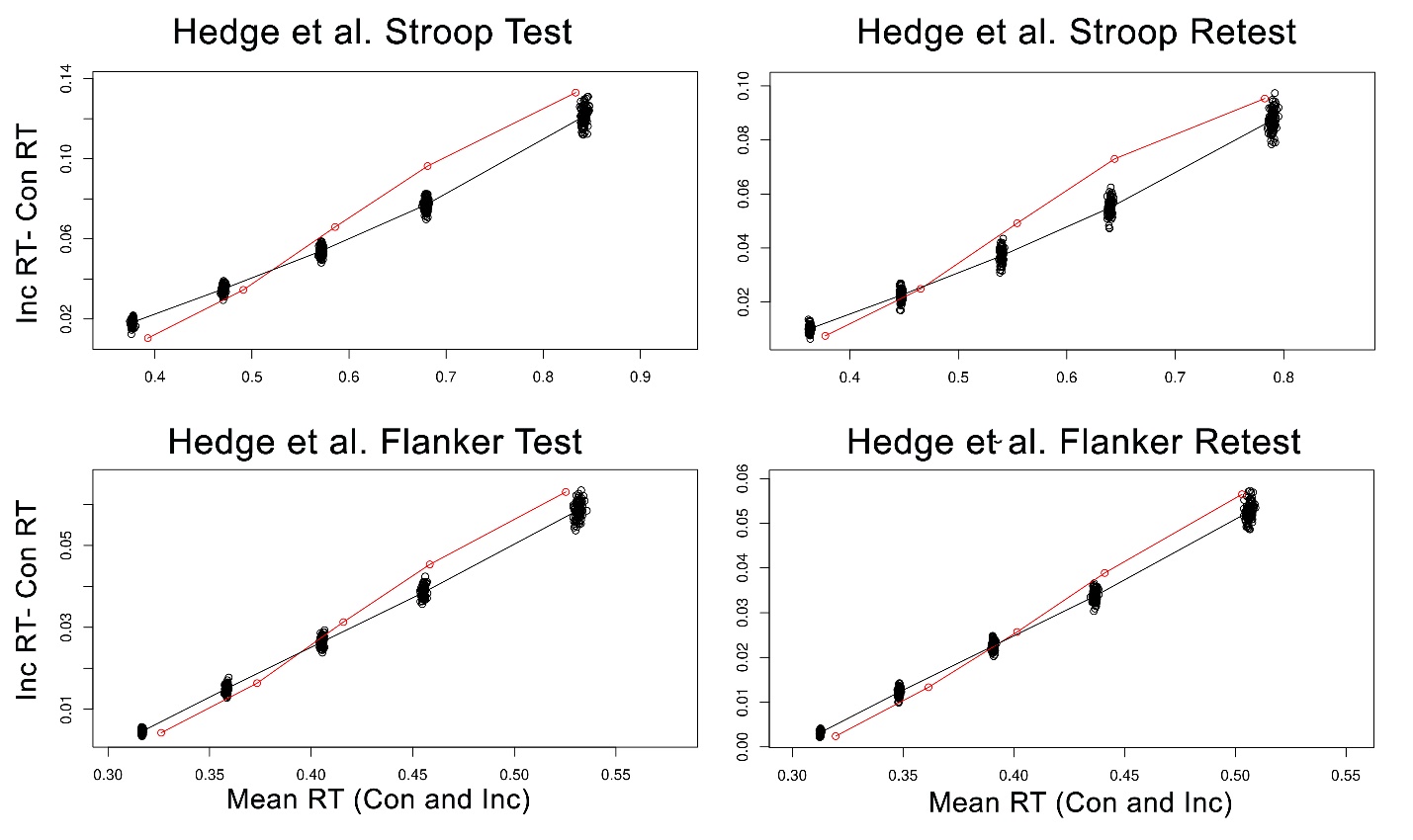
**

*Figure S8.* **Full Model Delta Function Fits for the Hedge et al. Dataset Stroop and Flanker Tasks.** Group-level delta functions representing the difference between congruent (Con) and incongruent (Inc) correct RT quantiles (.1, .3, .5, .7, and .9) on the y-axis against the congruent/incongruent mean on the x-axis, for empirical data (red points and lines) and data predicted by 100 posterior samples from the model (black points indicate the data predicted by each individual sample; black lines indicate the average of data predicted by all posterior samples).


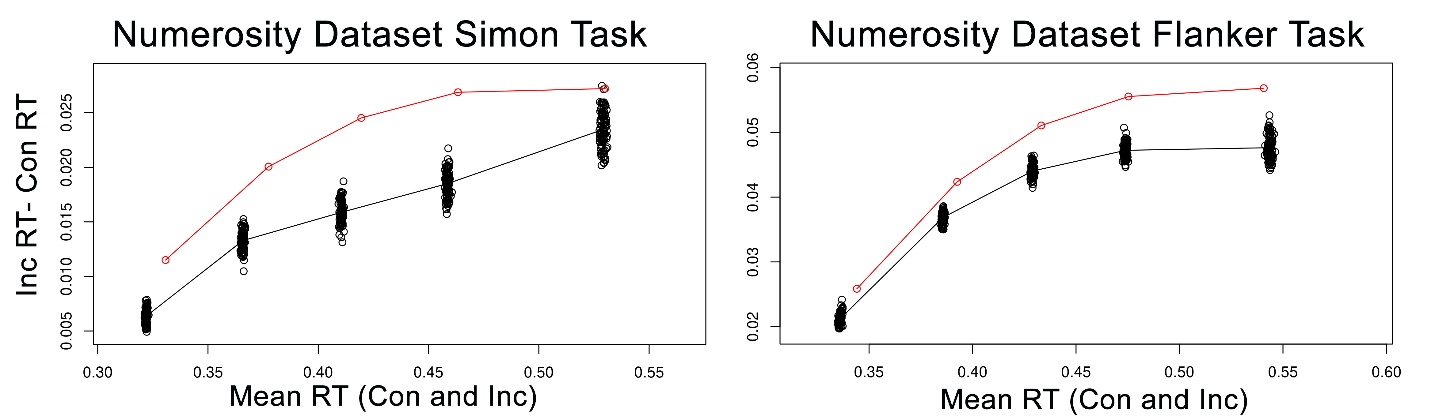


*Figure S9.* **Full Model Delta Function Fits for the Numerosity Dataset Simon and Flanker Tasks.** Group-level delta functions representing the difference between congruent (Con) and incongruent (Inc) correct RT quantiles (.1, .3, .5, .7, and .9) on the y-axis against the congruent/incongruent mean on the x-axis, for empirical data (red points and lines) and data predicted by 100 posterior samples from the model (black points indicate the data predicted by each individual sample; black lines indicate the average of data predicted by all posterior samples).

**
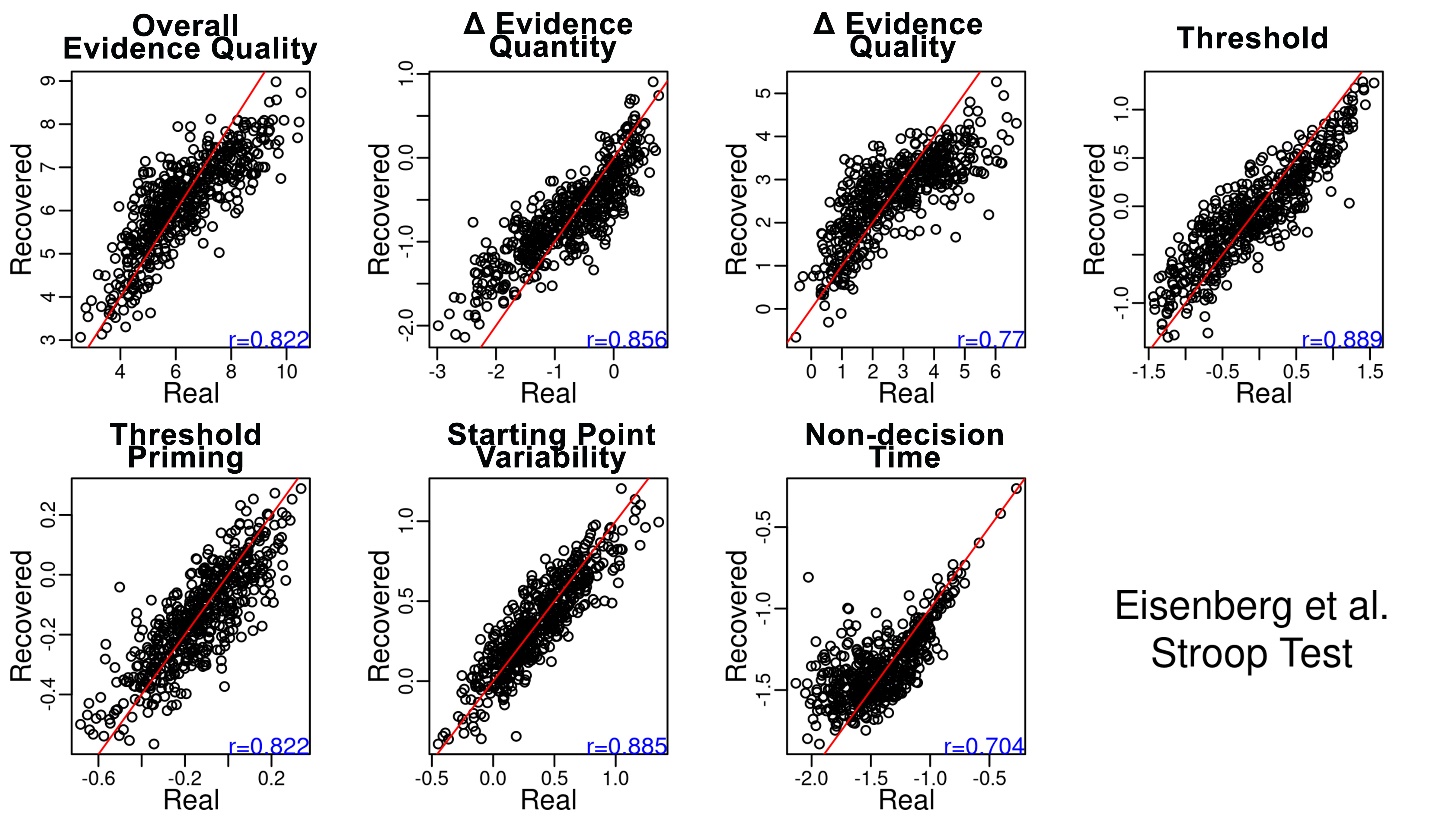
**

**Figure S10. Parameter Recovery Plots for the Eisenberg et al. Test Dataset Stroop Task.** Each point denotes an individual’s data-generating (real’) estimate (median of the individual-level posterior from the full model fit to observed data), versus their recovered estimate (median of the individual-level posterior from the full model fit to data simulated from the data-generating estimates). The red line indicates perfect recovery, and the blue text shows the correlation between the data-generating and recovered values for that parameter.


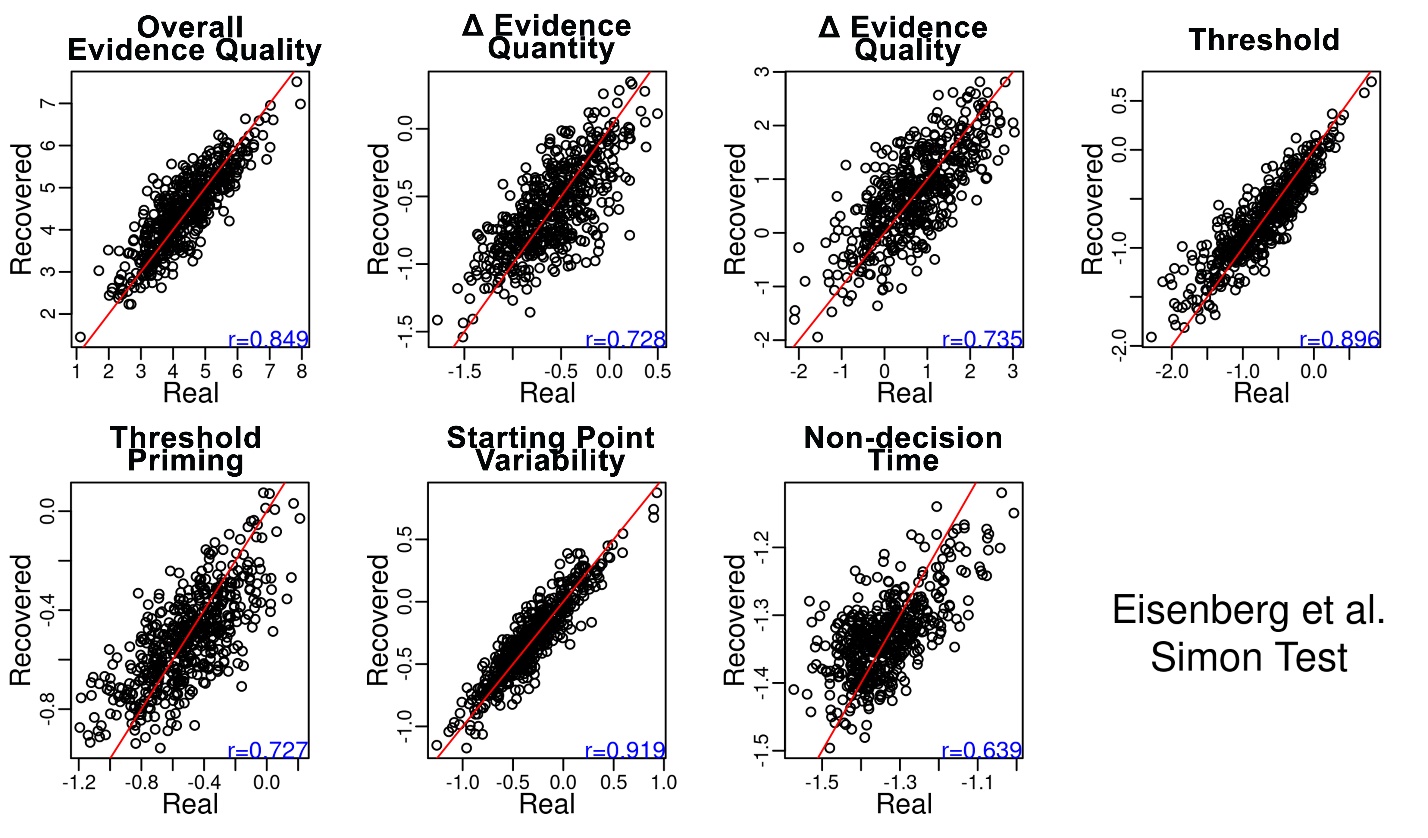


**Figure S11. Parameter Recovery Plots for the Eisenberg et al. Test Dataset Simon Task.** Each point denotes an individual’s data-generating (‘real’) estimate (median of the individual-level posterior from the full model fit to observed data), versus their recovered estimate (median of the individual-level posterior from the full model fit to data simulated from the data-generating estimates). The red line indicates perfect recovery, and the blue text shows the correlation between the data-generating and recovered values for that parameter.
